# Unpacking all-inclusive superordinate categories: Comparing correlates and consequences of global citizenship and human identities

**DOI:** 10.3389/fpsyg.2022.986075

**Published:** 2022-09-02

**Authors:** Margarida Carmona, Rita Guerra, John F. Dovidio, Joep Hofhuis, Denis Sindic

**Affiliations:** ^1^Instituto Universitário de Lisboa (ISCTE-IUL), Centro de Investigação e Intervenção Social, Lisbon, Portugal; ^2^Department of Psychology, Department of Social and Behavioral Sciences, Yale University, New Haven, CT, United States; ^3^Erasmus Research Center for Media, Communication, and Culture (ERMeCC), Erasmus University Rotterdam, Rotterdam, Netherlands

**Keywords:** all-inclusive superordinate identities, global citizenship identification, human identification, intergroup helping, autonomy-oriented help

## Abstract

Previous research suggests that all-inclusive superordinate categories, such as “citizens of the world” and “humans,” may represent different socio-psychological realities. Yet it remains unclear whether the use of different categories may account for different psychological processes and attitudinal or behavioral outcomes. Two studies extended previous research by comparing how these categories are cognitively represented, and their impact on intergroup helping from host communities toward migrants. In a correlational study, 168 nationals from 25 countries perceived the group of migrants as more prototypical of the superordinate category “citizens of the world” than their national group (relative outgroup prototypicality), whereas no differences in prototypicality occurred for the category “humans.” Identification with “citizens of the world” was positively associated with a disposition to oppose helping migrants and to offer dependency-oriented help. However, identification with “humans” was positively associated with helping in general, and with offering dependency- and autonomy-oriented help; and negatively associated with opposition to helping. The experimental study manipulated the salience of “citizens of the world” vs. “humans” vs. control category, among 224 nationals from 36 countries. Results showed that the salience of “humans” (vs. “citizens of the world”) triggered higher entitativity and essentialist perceptions, and dual-identity representations. No differences due to salience were found for representations of relative ingroup prototypicality or helping responses. Overall, these findings suggest that the interchangeable use of different labels is problematic, considering these might activate different representations, and thus, are likely to lead, in some circumstances, to different attitudinal or behavioral outcomes.

## Introduction

*Can humanity constitute an ingroup?* (Allport, 1954, p. 41-45)

Research on reducing intergroup conflict and improving intergroup relations has demonstrated the broad effectiveness of creating a sense of shared identity among people who originally conceived of themselves as members of different groups. For example, the main tenet of the common ingroup identity model (Gaertner and Dovidio, 2000) is that shifting the basis of categorization of “us vs. them” into a more inclusive “we” ameliorates intergroup tensions because it redirects the forces of ingroup favoritism to improve orientations toward others formerly perceived to be “them” (Gaertner and Dovidio, 2000; Gaertner et al., 2016). One implication of the robust evidence supporting this proposition is that *all-inclusive superordinate categories*—which encompass all human beings as a single group—may be uniquely effective for improving relations among diverse groups, because they create a sense of shared identity across multiple domains of difference (Barth et al., 2015). However, as Allport (1954) question about humanity implies and the current research investigates, the nature of an all-inclusive identity may influence the effectiveness of the common identity for improving intergroup relations. The present research examined whether and why alternative forms of all-inclusive identity might differ in effectiveness. We use the term ‘all-inclusive’ to refer to the highest level of identity abstraction (in categorical terms), which Allport (1954) specifies as the outer ring in his ‘circles of inclusion’ (Allport, 1954, p: 43), and not one related to a specified social context, such as national identity, or specific values or ideologies of inclusion. All-inclusive superordinate categories may be conceived as the broadest exemplar of recategorization into a common identity. Different constructs and categories have been proposed. Some focused on common humanity (e.g., *all humanity*, Barth et al., 2015), whereas others emphasized belongingness to a worldwide collection of people (e.g., *people all over the world*, McFarland et al., 2012; *world population*, Reese et al., 2016), or citizens (e.g., *citizens of the world*, ISSP, 2015; *global citizens*, Reysen and Katzarska-Miller, 2013). Overall, several positive impacts of identification with all-inclusive categories have been identified (e.g., increased prosocial behavior, McFarland et al., 2019; greater political solidarity from advantaged toward disadvantaged groups; Subašić et al., 2008). However, some detrimental effects have also been identified (e.g., weaker intentions to reduce global inequality; Reese et al., 2016). Recent discussions of these apparently inconsistent effects highlight the importance of considering the nature of these categories. Specifically, besides conveying shared identity, the specific meaning and cognitive representation of all-inclusive categories can vary, which can activate different processes and thus can have different intergroup consequences (Reysen and Katzarska-Miller, 2015; Reese et al., 2016; Carmona et al., 2020).

The main goal of the current research is to better understand how different all-inclusive superordinate categories may produce different effects and to illuminate the psychological processes that account for different outcomes. To do so we compared the categories *citizens of the world* and *humans*, considering their relationships to intergroup processes (Wenzel et al., 2016), cognitive representations (Gaertner et al., 2016), and impact on intergroup relations. We selected these two categories for comparison in the present work because they are frequently used in the largest cross-national surveys (e.g., EVS, 2020), as well as because they have attributes that may activate different psychological processes. For example, the category of *humans*, because it seems to activate more biological attributes (e.g., nature of the human species; appearance; need of bonding; Carmona et al., 2020), may influence perceptions of essentialism and motivate prosocial action toward others defined as human more than would the category of *citizens of the world*, which seems to activate more attitudinal attributes (e.g., multiculturalism; cosmopolitanism; Carmona et al., 2020, 2022).

According to the self-categorization theory (Turner et al., 1987), people cognitively represent ingroups, outgroups, or superordinate groups (i.e., groups inclusive of the ingroup and an outgroup) using category prototypes. These prototypes are composed of a fuzzy set of attributes that capture simultaneously perceived similarities within a particular group and differences between that group and other groups (Hogg and Smith, 2007). Different social groups have different prototypical content, which not only describes categories but also prescribes prototype-based attitudes and behaviors of group members (Turner and Reynolds, 2012).

The nature of superordinate categories may activate a variety of processes that can affect their impact. Ingroup projection is one such process. The ingroup projection model (Mummendey and Wenzel, 1999; Wenzel et al., 2007) focuses on the influence of relative ingroup prototypicality, which involves the tendency of people to use characteristics from their ingroups and familiar groups to define central, distinguishing characteristics of superordinate categories. The model proposes that when a superordinate category is salient and positively valued, members of a subgroup may “project” their ingroup’s attributes onto the prototype of the superordinate group. This projection process leads to relative ingroup prototypicality, in which people see their group as relatively more representative of the superordinate group than the outgroup. Because the outgroup is then perceived as less prototypical and therefore less normative, it is less valued.

Indeed, relative ingroup prototypicality, because it promotes ingroup favoritism as well as outgroup derogation and hostility, can undermine the positive effects of common ingroup identities (Wenzel et al., 2016)—including all-inclusive superordinate identities. For instance, citizens from a high-income country perceived their ingroup as more prototypical of the *world population* than the outgroup of citizens of lower-income countries (Reese et al., 2012; referred to by those authors as “developed” and “developing” countries”). The greater relative ingroup prototypicality was indirectly associated with less positive behavioral intentions toward the outgroup (Reese et al., 2012), along with weaker intentions to act against inequalities (Reese et al., 2016). Also, research on infrahumanization, which refers to beliefs that outgroup members possess fewer human characteristics than ingroup members, suggests that ingroup prototypicality may also be observed for human identity, as people tend to judge ingroup attributes as more human than those of the outgroup (Paladino and Vaes, 2009), and tend to create their concepts of “humanity” based on their impressions of their own group (Bilewicz and Bilewicz, 2012).

In the present research, we compared how the all-inclusive categories of *citizens of the world* and *humans* relate to relative ingroup prototypicality and to measures of intergroup helping (Studies 1 and 2) in a specific intergroup setting. We investigated how national citizens of a host country (ingroup) viewed and responded to migrants living in the same country (outgroup). We conceptualized national citizens as an advantaged group in numerical, economic, and social terms, in contrast to migrants who were conceived to be in a disadvantaged position in these terms. In Study 1, we examined this issue correlationally in terms of the strength of global citizenship identification (using the category *citizens of the world*) and human identification (using the category *humans*). In Study 2, we investigated this question experimentally in terms of the potentially different impacts of these forms of all-inclusive identification salient.

In both studies, we explored whether relative ingroup prototypicality would differ as a function of whether the category used was *citizens of the world* or *humans*. Ingroup projection is particularly likely to occur when a shared superordinate identity is made salient among members of a higher status group (Wenzel et al., 2007). Our participants were members of host communities, thus we expected relative ingroup prototypicality to occur in both studies. That is, we anticipated that participants would generally see their national ingroup’s characteristics, compared to those of migrants, as more prototypical of the perceived characteristics of *citizens of the world* and *humans*. We further considered the possibility that different perceptions of relative ingroup prototypicality with respect to the superordinate category may emerge because of the specific content that people may associate with what it means to be a *citizen of the world* and what it means to be a *human*. Previous research (Carmona et al., 2020) suggested a distinction between global citizenship-oriented labels (e.g., *citizens of the world*), which evoke aspects that people share as members of a global political community of citizens (e.g., cosmopolitan views), and humanness-oriented labels, which mainly evoke aspects that people share as members of the human species (e.g., physical appearance). Nonetheless, considering the lack of evidence examining relative ingroup prototypicality for *citizens of the world*, we acknowledge the exploratory nature of our research in this regard.

Besides exploring the potential degree to which relative ingroup prototypicality may be involved for these two all-inclusive superordinate categories, we examined the role of *citizens of the world* and *humans* on a particular type of intergroup helping—autonomy-oriented help (Studies 1 and 2). Autonomy-oriented help (e.g., providing the tools to solve a problem) is a form of assistance that is empowering because it fosters the capacity of others to achieve related goals independently in the future. Autonomy-oriented help contrasts with dependency-oriented help (e.g., providing the full solution to a problem), which involves assistance that establishes or reinforces the recipient’s continued need to rely on the benefactor. In Study 2, we also investigated how the two all-inclusive categories, *citizens of the world* and *humans*, are cognitively represented in terms of perceptions of entitativity, essentialism, and group representations. The theoretical background for considering these aspects is elaborated in the introduction of the individual studies.

### Social context

We focused our research on the context of migration because of the current practical and theoretical importance of this topic. Practically, international migration is occurring at an unprecedented pace, and it has generated considerable political and social controversy. In 2020, approximately 281 million people were living outside their country of origin, either by choice or by force (UN-United Nations, 2020), and face an increasingly hostile and polarized socio-political environment (Dempster and Hargrave, 2017; UNDP, 2020). Stronger restrictions to mobility are being put in place (e.g., physical walls at borders; surveillance control systems), and anti-immigration and xenophobic narratives are rising (Benedicto and Brunet, 2018; Bouron et al., 2021). A recent extreme example of these restrictions was the criminalization of helping migrants; in certain countries, one could face criminal charges for rescuing people at sea or offering food toward people on the move (AI-Amnesty International, 2019, 2020). Theoretically, several core social psychological processes (e.g., threat, discrimination) are prevalent and impactful in how host communities deal with migration (Verkuyten, 2018). A more comprehensive psychological understanding can offer insights that are valuable for achieving one of the United Nation’s Global Goals for 2030—*to empower and promote social*, *economic*, *and political inclusion of all people*. It is important to analyze pathways to build more inclusive societies globally, for example by fostering prosocial empowering interactions between host communities and migrants.

## Study 1

Study 1 investigated, correlationally, the extent to which the strength of global citizenship identification (using the category *citizens of the world*) and human identification (using the category *humans*) relate to relative ingroup prototypicality and intergroup help given by nationals citizens of a host country (ingroup) toward migrants (outgroup).

Intergroup helping might have different implications for intergroup power relations depending on the type of help given (Halabi and Nadler, 2017). Research inspired by the intergroup helping as status relations model (Nadler, 2002; Halabi et al., 2008) revealed that group members often engage in helping strategically to reinforce or establish a position of power over another group. Autonomy-oriented help reduces the recipient’s reliance on the benefactor in the future, and thus empowers those in need. By contrast, dependency-oriented help creates or reinforces the reliance of the recipient on the benefactor, which can maintain or widen the social disparity between the groups. Previous research demonstrated that, under conditions in which people view others in need as threatening in some way, people are more likely to offer dependency-oriented help to secure their advantageous social position (Halabi and Nadler, 2017). Of particular relevance to the current research, host-country members are generally less willing to offer migrants autonomy- than dependency-oriented help (Abad-Merino et al., 2013).

A common identity can affect the type of help that is exchanged between groups. Research on the common ingroup identity model (Gaertner and Dovidio, 2000; Gaertner et al., 2016) reveals that when a common identity is salient, people are generally more helpful to others formerly seen as members of an outgroup (Dovidio et al., 2008). Accordingly, all-inclusive identities have been generally related to prosocial outcomes (McFarland et al., 2019). In the present research, we hypothesized that having a stronger sense of global citizenship and human identification would be associated with greater helpfulness toward migrants. We further explored the possibility that those who strongly identify themselves with *citizens of the world* and *humans* would display either autonomy- as well as dependency-oriented helping toward migrants, or even different patterns. Previous research demonstrated, for example, that whereas members of one group were less willing to seek dependency- than autonomy-oriented help from another group when separate identities were salient, they were as likely to seek dependency- as autonomy-related help from the other group when common identity was salient (Halabi et al., 2014). Nonetheless, considering the lack of research examining these relationships with respect to help-giving, we refrained from offering directional hypotheses.

Overall, in Study 1, participants reported their level of global citizenship and human identification, along dimensions of self-definition and self-investment (Leach et al., 2008). The self-definition dimension involves individuals’ perceptions of themselves as similar to a group prototype, and of their group as sharing commonalities. The self-investment dimension relates to individuals’ positive feelings about and importance of their group membership, and their sense of belongingness. Then, we assessed relative ingroup prototypicality of national citizens compared to migrants, for *citizens of the world* and *humans*. Finally, participants responded to a variety of help-relevant measures.

To sum up, our hypotheses and exploratory aspects of Study 1 were as follows,

*H1*: Based on previous research (Wenzel et al., 2007), we hypothesized that participants would view the ingroup as relatively more prototypical than migrants of the all-inclusive category.

*H*2: Based on work on the common ingroup identity model (Gaertner et al., 2016), we hypothesized that global citizenship and human identification would be associated with greater helpfulness toward migrants.

We explored whether the degree of relative ingroup prototypicality would differ as a function of global citizenship or human identification and would be related to helping preferences and orientations.

We also explored whether these two forms of all-inclusive identity would relate to different tendencies for autonomy- and dependency-oriented help.

We tested these relationships controlling for general prosocial traits (social value orientation; Van Lange et al., 2007) and other general factors that predict orientations toward migrants—political orientation and national identification. Previous research has found that individuals who are more politically conservative (Manesi et al., 2019) and persons who have a stronger national identification (López et al., 2019) have more negative orientations toward migrants.

### Method

#### Participants and procedure

An *a priori* power analysis was conducted using G*Power (Faul et al., 2007). It indicated that a sample size of 80 participants would be required based on the predetermined parameters: effect size *f* = 0.20, power = 0.80, α = 0.05, 7 predictors. We oversampled in anticipation of non-valid responses. Participants were recruited *via* Mechanical Turk, in August and September 2019, and completed an online survey in Qualtrics platform, in exchange for monetary compensation (US$1.5). To be counted as part of the host community, individuals and their parents had to be living in their and their parents’ country of birth and hold citizenship (to minimize the possibility that participants would conceive of themselves as migrants). To minimize forged responses, multiple validation procedures were implemented (i.e., robot check, control questions, and open-answers screening). The full protocol is available in Supplementary material.

We complied with APA Ethical Principles of Psychologists and Code of Conduct (APA-American Psychological Association, 2017), and the Code of Ethical Conduct in Research in place at the first author’s institution. All participants were 18 or older; informed consent was requested, and participants were debriefed. The informed consent was completed by 315 participants. However, 45 responses were excluded because they did not conform to the inclusion criterion about residency and citizenship in the host society. An additional 102 individuals were excluded because they failed validation procedures. The final sample included 168 participants, who had sufficient proficiency in English, from 25 different countries (mostly United States, Brazil, United Kingdom, and India; details in Supplementary material). The mean age was 32.11 years (*SD* = 8.2, range: 18–58), 66.1% participants identified as a man and 33.9% as a woman; 78% had higher education; 69% were employed. Participants displayed heterogeneous political views (*M* = 4.02, *SD* = 1.82, range: 1–7, n = 155): 40.5% positioned themselves at the left/center-left; 35.7% at the right/center-right and 16.1% at the center.

Participants indicated their country of birth and residence, and nationality. Then, the measures were administered in the following order^1^: group identification (i.e., global citizenship identification, human identification, and national identification, in a randomized order); general prosociality (altruistic orientation), relative ingroup prototypicality for *citizens of the world* and for *humans*, in a randomized order; and helping preferences and helping orientations toward migrants. Sociodemographic information was collected at the end, and participants were thanked and debriefed.

### Materials

All items within each scale were presented in a randomized order and were measured using a 7-point Likert-type scale (1 = *strongly disagree* to 7 = *strongly agree*) unless stated otherwise.

#### Group identification

Group identification was assessed by the Multicomponent Ingroup Identification Scale by Leach et al. (2008) and was administered three times: global citizenship identification (using the label *citizen of the world*), human identification (using the label *humans*), and national identification (using participant’s reported nationality). Global citizenship identification and human identification were predictors of primary interest. National identification was included because it is generally related to negative orientations toward migrants (López et al., 2019); it was treated as a covariate in the analyses. The self-definition dimension (4 items; *α_c.world_* = 0.86; *α_human_* = 0.83; *α_national_* = 0.85) assessed self-stereotyping (e.g., “I have a lot in common with the average *citizen of the world/human/national*”) and ingroup homogeneity (e.g., “*Citizens of the world/humans/nationals* are very similar to each other”). The self-investment dimension (10 items; *α_c.world_* = 0.94; *α_human_* = 0.90; *α_national_* = 0.94) assessed satisfaction with the membership (e.g., “Being *a citizen of the world/a human/nationality* gives me a good feeling”), centrality of group membership (e.g., “The fact that I am *a citizen of the world/a human/nationality* is an important part of my identity”), and solidarity with other group members (e.g., “I feel solidarity with *citizens of the world/humans/nationals*”). An exploratory factor analysis^2^ showed a clear distinction between national identification, and the self-definition and self-investment dimensions of global citizenship and human identification. For this reason, in the following analyses, we treated them separately.

#### Altruistic orientation

Altruistic orientation which represented individual differences in prosociality generally and was entered as a covariate in the analyses, was measured using the 6 primary items of the Social Value Orientation (SVO) Slider Measure (Murphy et al., 2011). For each item, participants allocated points that supposedly would be converted into real money, between themselves and a non-identified person. This measure provides a continuous angle representing the ratio of allocations to oneself versus another person, that can be computed categorically to identify four types of social orientations. Higher values of SVO angle refer to altruistic (>57.15°) and prosocial individuals (22.45° to 57.15°), whereas lower values refer to individualistic (−12.04° to 22.45°) and competitive (< −12.04°) individuals. Previous research has shown good psychometric properties of the measure (Murphy et al., 2011).

#### Relative ingroup prototypicality

Relative ingroup prototypicality was measured by adapting from Wenzel et al. (2003; Study 3). Participants typed three attributes they considered characteristic of their national group (ingroup) compared to migrants (outgroup), and three attributes they considered characteristic of migrants compared to their national group. The 6 self-generated attributes were randomly presented, and participants rated to what extent each attribute applies to *citizens of the world* and *humans* (i.e., the scale was administered twice adapting the target group; 1 = *Does not apply at all to citizens of the world/humans*, 7 = *Applies very much to citizens of the world/humans*). Relative ingroup prototypicality (RIP) for *citizens of the world* and *humans* was computed as the difference score between the mean typicality ratings of ingroup attributes and the mean typicality ratings of outgroup attributes. Positive scores indicate that participants perceived ingroup (national group) attributes’ as more prototypical of the superordinate categories than those of the outgroup (migrants), that is, RIP. Correspondingly, negative scores indicate that participants perceived migrants’ attributes as more prototypical than those of their national group, that is relative outgroup prototypicality.

#### Helping preferences

Helping preferences assessed which helping response nationals prefer to offer toward migrants in helping situations that could occur naturalistically and were measured using 10 scenarios adapted from Halabi et al. (2008). Participants were presented with a cover story in a short video informing that a new international website was launched, where migrants can chat with nationals to ask them for help in finding solutions to problems they encounter daily. Participants were told that they would be presented with different problems and a list of possible solutions, and that they would be asked to select the best solution to be recommended to future users of the website. Then, the 10 scenarios were randomly presented, covering diverse problems in different contexts (e.g., make an appointment in a health facility, create a resumé to apply to a job, obtain a residence permit). Participants were asked to select one out of four possible actions: (1) provide a full solution to the problem – dependency-oriented response (e.g., “contact the health facility and make the appointment for the migrant user”); (2) provide instructions to solve the problem—autonomy-oriented response (e.g., “inform and support the migrant user on how to identify a health facility and how to make an appointment”); (3) no help (e.g., “*national* user should not help, because the migrant user should find a solution to this problem on his/her own”); (4) none of the previous options should be recommended. As confirmed by a multiple correspondence analysis,^3^ participants tended to display patterns of preferences for dependency- or autonomy-oriented responses, independently of the scenario’s content. We computed two measures based on the helping responses for each scenario. First, to measure participants’ preference for choosing to help migrants independently of the type of help given, *preference for helping in general* was computed as the count of the number of times dependency-oriented and autonomy-oriented responses were chosen, ranging from 0 (no helping options were selected) to 10 (in all 10 scenarios participants choose to offer either dependency-oriented or autonomy-oriented help). Second, to measure their preference for a specific type of help response, *preference for autonomy- relative to dependency-oriented help* referred to the proportion of times when help was given that participants recommended an autonomy-oriented response (i.e., computed as the number of times autonomy-oriented responses were selected divided by the *preference for helping in general*). This measure ranged from 0 (when help was given, no autonomy-oriented responses were chosen) to 1.00 (when help was given, in all scenarios, participants recommended an autonomy-oriented response as the best solution to the problem).

#### Helping orientations

Helping orientations were measured by the Helping Orientations Inventory (Maki et al., 2017) to assess participants’ individual dispositions to help migrants, namely *orientation for dependency-oriented help* (5 items, e.g., “In general, solving migrants’ problems for them is good for society because it helps meet immediate needs”; *α*_dependency_ = 0.76),^4^ *orientation for autonomy-oriented help* (8 items, e.g., “Teaching migrants to take care of themselves is good for society because it makes them independent”; *α*_autonomy_ = 0.88), and a general *orientation for opposition to helping* (8 items, e.g., “Helping migrants only makes them more needy in the future”; *α*_opposition_ = 0.93).

### Results

Means, *SD*s, and zero-order correlations for the main variables are presented in Table 1. A full table including secondary variables is available in Supplementary material.

**Table 1:** Means, *SD*s, and zero-order correlations among main variables.

	1	2	3	4	5	6	7	8	9	10	11	12	13
1. Global ident.: Self-investment	–												
2. Human ident: Self-investment	0.71^**^												
3. Global ident.: Self-definition	0.65^**^	0.56^**^											
4. Human ident: Self-definition	0.39^**^	0.46^**^	0.62^**^										
5. Pref. for helping in general	0.21^**^	0.26^**^	0.17^*^	0.25^**^									
6. Orient. to opposition to helping	0.01	0.02	0.07	−0.06	−0.44^**^								
7. Orientation for dependency	0.37^**^	0.32^**^	0.35^**^	0.34^**^	0.29^**^	0.13							
8. Preference for autonomy	−0.09	−0.03	−0.07	−0.07	0.18^*^	−0.39^**^	−0.35^**^						
9. Orientation for autonomy	0.32^**^	0.34^**^	0.22^**^	0.29^**^	0.62^**^	−0.28^**^	0.40^**^	0.17^*^					
10. National identification	0.44^**^	0.62^**^	0.37^**^	0.31^**^	0.04	0.25^**^	0.26^**^	−0.09	0.16^*^				
11. Altruistic orientation	0.07	0.03	−0.01	−0.05	0.20^**^	−0.25^**^	−0.03	0.09	0.19^*^	−0.13			
12. RIP for citizens of the world	0.04	0.10	0.11	−0.01	−0.07	0.24^**^	−0.04	0.02	−0.14	0.21^**^	−0.18^*^		
13. RIP for humans	0.13	0.12	0.18^*^	0.13	0.10	0.03	0.10	−0.09	0.10	0.11	−0.10	0.42^**^	
14. Political orientation	−0.09	−0.03	−0.09	−0.08	−0.43^**^	0.59^**^	0.04	−0.35^**^	−0.36^**^	0.22^**^	−0.20^*^	0.16^*^	0.07
***M***	4.92	5.24	4.63	5.16	8.91	3.09	4.38	0.83	5.51	4.99	26.88	−0.62	−0.09
***SD***	1.15	1.07	1.26	1.17	2.14	1.45	1.13	0.20	0.96	1.20	13.58	1.48	1.37

*p < 0.05; **p < 0.01.

As presented in Table 1, the self-investment and self-definition dimensions were moderate to highly correlated within and between both global citizenship and human identification; were positively associated with national identification, but not with altruistic orientation or political orientation; and, were positively related to a *preference helping in general*, as well as *orientation for autonomy-oriented help* and *for dependency-oriented help* toward migrants, but not to a *preference for autonomy- relative to dependency-oriented help*. Moreover, altruistic orientation was positively related to *preference for helping in general* and *orientation for autonomy-oriented help*, and negatively to *orientation for opposition to helping*. Whereas political orientation (higher values indicate a right-wing orientation) was positively related to *orientation for opposition to helping*, and negatively to *preference for helping in general*, as well as to *preference* and *orientation for autonomy-oriented help*.

#### Relative ingroup prototypicality

We examined the degree to which participants projected their ingroup’s attributes onto the two all-inclusive superordinate categories (*citizens of the world* and *humans*) relative to the outgroup’s (migrants’ attributes)—relative ingroup prototypicality (RIP)^5^. We also tested the relationship between the degree to which participants endorsed each of the all-inclusive forms of identification (global citizenship and human identification) and their levels in RIP for the respective categories (i.e., *citizens of the world* and *humans*, respectively). In addition, we explored the relationships between RIP for *citizens of the world* and *humans*, separately, and the specific intergroup helping measures.

In terms of the overall degree to which participants exhibited RIP for *citizens of the world* (*M* = −0.62, *SD* = 1.48) and for *humans* (*M* = −0.09, *SD* = 1.37) both showed negative means. Negative scores indicate that, contrary to our expectations (H1), participants perceived migrants’ attributes as more (not less) prototypical of *citizens of the world* and *humans* than those of their national ingroup, producing relative *outgroup* prototypicality (ROP) instead of RIP. However, one-sample *t*-tests revealed that this ROP effect was significantly different from zero only for *citizens of the world*, *t*(166) = −5.429, *p* < 0.001, and not for *humans*, *t*(166) = −0.864, *p* = 0.389. Indeed, a paired sample *t*-test showed that the two means were significantly different from each other, *t*(166) = −4.448, *p* < 0.001. In sum, participants considered migrants as more prototypical of *citizens of the world* than their national group members, whereas neither RIP nor ROP were observed for *humans*.

As presented in Table 1, stronger endorsement of global citizenship identification was not significantly related to RIP for *citizens of the world* either measured as self-investment or self-definition. Similarly, human identification representing self-investment and self-definition were not significantly related to RIP for *humans*. The analyses did reveal significant relationships between RIP for *citizens of the world* and the measures of intergroup helping. Specifically, RIP for *citizens of the world* was not significantly related to any of the measures involving helping migrants, except for participants exhibiting stronger RIP for *citizens of the world* which were more opposed to helping migrants. RIP for *humans* was not significantly related to any of the measures involving helping migrants.

#### Predicting helping in general (independently of the type)

In this set of analyses, we explored the main predictors of primary interest—global citizenship and human identification (in terms of self-investment and self-definition)—on measures of helping, regardless of the type of help given to migrants. Specifically, we report the results for helping preferences (i.e., *preference for helping in general*, as assessed by scenarios) and orientations (i.e., *orientation for opposition to helping*, as assessed by Helping Orientations Inventory, Maki et al., 2017). In these analyses, we control for altruistic orientation to distinguish between general tendencies and the tendency to help migrants specifically, and for national identification, political orientation and RIP to help isolate the effects of global citizenship and human identification.

We conducted four hierarchical multiple regressions (Table 2), two for *preference for helping in general* (models 1, 2, and 3) and two for *orientation for opposition to helping* (models 4, 5, and 6). Control variables were included in the first step of hierarchical multiple regressions for each outcome (model 1—*preference for helping in general*, and model 4—*orientation for opposition to helping*): altruistic orientation, political orientation, RIP for *citizens of the world* and *humans*, and national identification (treated as a unidimensional variable). Identification with *citizens of the world* and *humans* were included in the second step, separately for self-investment (models 2 and 5) and self-definition dimensions (models 3 and 6).

**Table 2:** Hierarchical multiple regression results for helping preferences and orientations regardless of the type of help.

	Preference for helping in general						
		95% CI for *B*					
	*B*	*LL*	*UL*	*SE B*	*β*	*R* ^2^	Δ*R*^2^
**Model 1**						0.25	0.22^***^
Constant	8.75	7.06	10.45	0.86			
Altruistic orientation	0.02^+^	0.00	0.05	0.01	0.13^+^		
Political orientation	−0.53^***^	−0.70	−0.35	0.09	−0.43^***^		
RIP for citizens of the world	−0.13	−0.37	0.11	0.12	−0.09		
RIP for humans	0.29^*^	0.04	0.55	0.13	0.18^*^		
National identification	0.31^*^	0.03	0.58	0.14	0.16^*^		
**Model 2 (Self-investment)**						0.29	0.25^*^
Constant	7.59^***^	5.66	9.53	0.98			
Altruistic orientation	0.02^+^	0.00	0.04	0.01	0.12^+^		
Political orientation	−0.48^***^	−0.66	−0.30	0.09	−0.40^***^		
RIP for citizens of the world	−0.12	−0.35	0.12	0.12	−0.08		
RIP for humans	0.25^*^	0.00	0.51	0.13	0.15^*^		
National identification	0.01	−0.34	0.36	0.18	0.00		
Global citizenship ident: SI	−0.16	−0.54	0.23	0.19	−0.08		
Human identification: SI	0.63	0.16	1.10	0.24	0.30^**^		
**Model 3 (Self-definition)**						0.29	0.26^*^
Constant	7.20^***^	5.19	9.22	1.02			
Altruistic orientation	0.02	0.00	0.05	0.01	0.15^*^		
Political orientation	−0.49^***^	−0.67	−0.31	0.09	−0.40^***^		
RIP for citizens of the world	−0.06	−0.30	0.18	0.12	−0.04		
RIP for humans	0.21	−0.04	0.47	0.13	0.13		
National identification	0.16	−0.13	0.46	0.15	0.09		
Global citizenship ident.: SD	−0.11	−0.43	0.21	0.16	−0.06		
Human identification: SD	0.50	0.14	0.86	0.18	0.26^**^		
	**Orientation for opposition to helping**						
**Model 4**						0.40	0.38^***^
Constant	1.05^*^	0.03	2.07	0.52			
Altruistic orientation	−0.01	−0.03	0.00	0.01	−0.11		
Political orientation	0.43^***^	0.32	0.54	0.05	0.53^***^		
RIP for citizens of the world	0.13	−0.02	0.27	0.07	0.13^+^		
RIP for humans	−0.08	−0.23	0.08	0.08	−0.07		
National identification	0.14	−0.02	0.31	0.08	0.11^+^		
**Model 5 (Self-investment)**						0.40	0.37
Constant	1.08^+^	−0.11	2.27	0.60			
Altruistic orientation	−0.01	−0.03	0.00	0.01	−0.11		
Political orientation	0.43^***^	0.32	0.54	0.06	0.53^***^		
RIP for citizens of the world	0.13	−0.02	0.27	0.07	0.13^+^		
RIP for humans	−0.08	−0.23	0.08	0.08	−0.07		
National identification	0.17	−0.05	0.39	0.11	0.13		
Global citizenship ident.: SI	0.13	−0.11	0.36	0.12	0.10		
Human identification: SI	−0.15	−0.44	0.14	0.15	−0.11		
**Model 6 (Self-definition)**						0.43	0.40^*^
Constant	1.43^*^	0.22	2.64	0.61			
Altruistic orientation	−0.01	−0.03	0.00	0.01	−0.12^+^		
Political orientation	0.43^***^	0.32	0.54	0.05	0.53^***^		
RIP for citizens of the world	0.09	−0.05	0.23	0.07	0.09		
RIP for humans	−0.06	−0.21	0.10	0.08	−0.05		
National identification	0.15	−0.03	0.33	0.09	0.12^+^		
Global citizenship ident.: SD	0.24	0.05	0.44	0.10	0.21^*^		
Human identification: SD	−0.30	−0.51	−0.08	0.11	−0.23^**^		

Model = “Enter” method in SPSS Statistics; B = unstandardized regression coefficient; CI = confidence interval; LL = lower limit; UL = upper limit; SE B = standard error of the coefficient; β = standardized coefficient; R^2^ = coefficient of determination; ΔR^2^ = adjusted R^2^. SI: Self-investment; SD: Self-definition.

* *p* < 0.05;

** *p* < 0.01;

*** *p * < 0.001;

+ *p* < 0.10.

As presented in Table 2, regarding *preference for helping in general*, the full model for self-investment (i.e., including all covariates and self-investment dimensions—model 2) was statistically significant (*R*^2^ = 0.287, *F*[7, 146] = 8.381, *p* < 0.001; adjusted *R*^2^ = 0.252), and the addition of self-investment with *citizens of the world* and *humans* led to a statistically significant increase in *R^2^* of 0.039, *F*(2, 146) = 4.019, *p* = 0.020. However, only self-investment as *humans* was associated with a higher *preference for helping in general*, over and above the significant negative effect of political orientation and the positive effect of relative ingroup prototypicality for *humans*. Similarly, the full model for self-definition (i.e., including all covariates and self-definition dimensions; model 3) was statistically significant (*R*^2^ = 0.290, *F*[7, 146] = 8.514, *p* < 0.001; adjusted *R*^2^ = 0.256), and the addition of self-definition as *citizens of the world* and *humans* led to a statistically significant increase in *R^2^* of.042, *F*(2, 146) = 4.369, *p* = 0.014. Again, only self-definition as *humans* was associated with a higher *preference for helping in general*, over and above the significant negative effect of political orientation and the positive effect of altruistic orientation. Contrary to the expected (H2), self-investment and self-definition as *citizens of the world* were not associated with a *preference for helping in general*.

Regarding *orientation for opposition to helping*, the full model for self-investment (i.e., including all covariates and self-investment dimensions; model 5) was statistically significant, *R*^2^ = 0.402, *F*(7, 146) = 14.013, *p* < 0.001; adjusted *R*^2^ = 0.373; however, the addition of self-investment with *citizens of the world* and *humans* did not significantly increase explained variance, *R^2^* of 0.006, *F*(2, 146) = 0.702, *p* = 0.497. Self-investment as *citizens of the world* and *human* were not associated with *orientation for opposition to helping* migrants; only political orientation showed a significant positive effect. On the contrary, the full model for self-definition (i.e., including all covariates and self-definition dimensions; model 6) was statistically significant, *R*^2^ = 0.431, *F*(7, 146) = 15.811, *p* < 0.001; adjusted *R*^2^ = 0.404, and the addition of self-definition as *citizens of the world* and *humans* led to a statistically significant increase in *R^2^* of 0.035, *F*(2, 146) = 4.502, *p* = 0.013. However, whereas self-definition as a *citizen of the world* was positively associated with *orientation for opposition to helping*, self-definition as a *human* was negatively associated, over and above the significant positive effect of political orientation.

#### Predicting dependency and autonomy-oriented help

In this last set of analyses, we explored the main predictors of primary interest—global citizenship and human identification—on the type of help given to migrants. Specifically, we report the results for helping orientations (i.e., *orientation for dependency-oriented help* and *for autonomy-oriented help*); results for *preference for autonomy- relative to dependency-oriented help* (as assessed by scenarios) did not reveal significant effects and are presented in Supplementary material. In these analyses, we only control for political orientation, which showed a consistent relationship with helping in the previous analysis.

We conducted four multiple regressions (Table 3) for *orientation for dependency-oriented help* (models 1 and 2) and *orientation for autonomy-oriented help* (models 3 and 4), separately for self-investment and self-definition dimensions of global citizenship and human identification.

**Table 3:** Multiple regressions result for types of help.

	Orientation for dependency						
		95% CI for *B*					
	*B*	*LL*	*UL*	*SE B*	β	*R* ^2^	Δ*R*^2^
**Model 1 (Self-investment)**						0.14	0.13
Constant	2.18^***^	1.22	3.15	0.49			
Political orientation	0.05	−0.05	0.14	0.05	0.07		
Global citizenship ident.: SI	0.29^**^	0.08	0.50	0.11	0.29^**^		
Human identification: SI	0.11	−0.12	0.35	0.12	0.11		
**Model 2 (Self-definition)**						0.16	0.14
Constant	2.12^***^	1.20	3.03	0.46			
Political orientation	0.05	−0.04	0.14	0.05	0.08		
Global citizenship ident.: SD	0.20^*^	0.03	0.37	0.09	0.22^*^		
Human identification: SD	0.22^*^	0.03	0.41	0.10	0.22^*^		
	**Orientation for autonomy**						
**Model 3 (Self-investment)**						0.25	0.23
Constant	4.56^***^	3.78	5.33	0.39			
Political orientation	−0.18^***^	−0.26	−0.11	0.04	−0.35^***^		
Global citizenship ident.: SI	0.06	−0.11	0.23	0.09	0.07		
Human identification: SI	0.27^**^	0.08	0.45	0.09	0.29^**^		
**Model 4 (Self-definition)**						0.22	0.20
Constant	4.92^***^	4.16	5.68	0.38			
Political orientation	−0.18	−0.26	−0.10	0.04	−0.34^***^		
Global citizenship ident.: SD	0.01	−0.13	0.15	0.07	0.01		
Human identification: SD	0.25^**^	0.09	0.40	0.08	0.29^**^		

Model = “Enter” method in SPSS Statistics; B = unstandardized regression coefficient; CI = confidence interval; LL = lower limit; UL = upper limit; SE B = standard error of the coefficient; β = standardized coefficient; R^2^ = coefficient of determination; ΔR^2^ = adjusted R^2^. SI: Self-investment; SD: Self-definition.

* *p* < 0.05;

** *p* < 0.01;

*** *p* < 0.001;

+ *p * < 0.10.

As presented in Table 3, regarding *orientation for dependency-oriented help*, the model for self-investment (model 1) was statistically significant (*R*^2^ = 0.143, *F*[3, 151] = 8.411, *p* < 0.001; adjusted *R*^2^ = 0.126), and only self-investment as *citizens of the world* was positively related to *orientation for dependency-oriented help*, whereas self-investment as *humans* was not. The model for self-definition dimensions (model 2) was statistically significant (*R*^2^ = 0.159, *F*[3, 151] = 9.541, *p* < 0.001; adjusted *R*^2^ = 0.143), and both self-definition as a *citizen of the world* and as a *human* were positively related to *orientation for dependency-oriented help*. Political orientation was not related to *orientation for dependency-oriented help*.

Regarding *orientation for autonomy-oriented help*, the model for self-investment (model 3) was statistically significant (*R*^2^ = 0.249, *F*[3, 151] = 16.702, *p* < 0.001; adjusted *R*^2^ = 0.234). Only self-investment with *humans* was positively related to *orientation for autonomy-oriented help*, whereas self-investment with *citizens of the world* was not. Similarly, the model for self-definition (model 4) was statistically significant (*R*^2^ = 0.216, *F*[3, 151] = 13.841, *p* < 0.001; adjusted *R*^2^ = 0.200), and only self-definition as a *human* was positively related to *orientation for autonomy-oriented help*. Political orientation was negatively associated with *orientation for autonomy-oriented help*, in both models.

### Discussion

Study 1 revealed that global citizenship and human identification were moderately to strongly related. However, consistent with the proposition that the specific content of an all-inclusive category is also important, these forms of identification also related distinctively to intergroup outcomes. For instance, consistent with work on the common ingroup identity model (Gaertner and Dovidio, 2000), although global citizenship and human identification both had significant positive correlations with preference for helping migrants generally, when considered as predictors and controlling for other relevant variables (e.g., political orientation, altruistic orientation) identification as *humans* was a significant predictor while identification as *citizens of the world* was not. Greater identification as *humans* was related to less opposition to helping migrants (albeit significantly only for self-definition). By contrast, greater identification as *citizens of the world* was positively (only for self-definition) related to opposition to helping.

The findings concerning *opposition to help* are surprising considering that are not in line with research showing that endorsing an all-inclusive identity improves prosocial orientations (McFarland et al., 2019). But, before providing possible explanations for these results in the General Discussion, a specific limitation should be addressed herein. We should note that the items assessing *orientation for opposition to helping* highlighted the negative outcomes of helping (e.g., “Solving migrants’ problems for them makes their situation worse in the long run”; Maki et al., 2017). To our understanding, these items do not merely reflect that “people are simply opposed to helping others” (Maki et al., 2017, p. 690), but might also reflect a concern about or the rejection of the undesirable outcomes of helping. For this reason, participants’ interpretation of these items is not clear, and further studies are needed.

Moreover, the more participants identified themselves as *citizens of the world*, the higher their orientation to offer dependency-oriented help toward migrants; whereas the more they identified as *humans*, the higher their orientation to offer either dependency- or autonomy-oriented help toward migrants. Overall, this pattern of findings supports the proposal that stronger all-inclusive orientations relate to more positive orientations to migrants but also suggests the promise of distinguishing how different all-inclusive identities may have different effects.

The manner by which identification as *humans* or as *citizens of the world* may have different effects is not clearly documented in Study 1. One of the factors we considered, relative ingroup prototypicality (RIP, Wenzel et al., 2007) did not appear to play a systematic role. Unexpectedly, we generally observed a relative outgroup prototypicality effect, not relative ingroup prototypicality. Also, the strength of global citizenship and human identification did not significantly predict RIP for *citizens of the world* and for *humans*, respectively. RIP for *citizens of world* did have significant, positive zero-order correlation with opposition to helping migrants, but it did not have a significant negative correlation with helping. RIP for *humans* did not significantly correlate with either helping measures. In addition, participants showed no systematic differences as a function of identification or RIP in autonomy- compared to dependency-oriented helping.

We note, however, that Study 1 was correlational and, while we measured and controlled for a range of relevant effects (e.g., political orientation, altruistic orientation, national identification), unmeasured variables might still be operative in ways that obscured the potential effects of our main variables of interest. Study 2 was therefore designed as an experiment.

## Study 2

Study 2 investigated, experimentally, the potentially different impacts of making *citizens of the world* and *humans* salient on relative ingroup prototypicality and intergroup help given by national citizens toward migrants. As in Study 1, we distinguished between autonomy and dependency-oriented help. Additionally, we explored the perceptions about entitativity and essentialism that may be elicited by the two categories, and how the different subgroups are represented within these common identities (one-group or dual-identity group representations), as these aspects may shape intergroup dynamics.

Entitativity represents the perception of the “groupness” of a social category (i.e., members’ similarities, interaction, common goals, fate, and the importance given to it). Essentialism describes the degree to which a category is perceived as natural, immutable and historically persistent, in which members are bonded by an underlying, often biological, essence (Lickel et al., 2000; Hamilton et al., 2004; Demoulin et al., 2006; Haslam, 2017). Importantly, people are inclined to develop stereotypic judgments about social categories when it is highly essentialized and tend to have polarized impressions when they perceive a group as highly entitative (Hamilton et al., 2004). Essentialist beliefs have been associated with several negative intergroup outcomes (e.g., prejudice; less interaction with essentialized outgroup members; resistance to egalitarian intergroup relations; strategic use of essentialism beliefs to exclude others from group membership; negative bias toward immigrants; Haslam et al., 2002; Bastian and Haslam, 2008; Morton et al., 2009; Pehrson et al., 2009; Haslam, 2017). Even though social categories are not conceived as highly entitative or homogenous, they tend to be essentialized (e.g., Karasawa et al., 2019), particularly those that have a biological basis (Hamilton et al., 2004). Previous research has suggested that the category *humans* might be highly essentialized (e.g., Haslam et al., 2005). We aim to explore whether *citizens of the world* might be perceived as less essentialized and more entitative than *humans*, considering their differences in meaning. Being a human being has strong biological connotation; whereas *citizens of the world* seems to activate less biological-related content, as it tends to describe individuals who hold a beyond-nation scope of concern and responsibility (Carmona et al., 2022).

Second, making common identity salient can alter the way people cognitively represent groups through the process of recategorization that changes an initial representation of different groups as separate groups to a shared identity, either as one single group emphasizing similarities among members (i.e., one-group representation) or as two subgroups on the same team, which recognizes and values both similarities and differences between subgroups (i.e., dual-identity representation; Gaertner and Dovidio, 2000). Research has shown that both types of shared-identity representations reduce prejudice and facilitate prosocial intergroup behavior toward former outgroup members (Dovidio et al., 2010; Gaertner et al., 2016). We investigated whether making the all-inclusive identity as *citizens of the world* or as *humans* salient would differ in the degree to which members of host communities adopt a one-group or a dual-identity representation inclusive of migrants. Previous research has shown that making shared identity salient is less likely to produce a one-group representation when it activates the need of members to differentiate and reaffirm their original, different group identities (Crisp et al., 2006). Also, the effectiveness of dual-identity representation might be weakened because when intergroup differences are highlighted, subgroup members may regard their subgroup’s attributes as more prototypical of the common category (i.e., ingroup projection; Gaertner et al., 2016). Like other forms of common identities, all-inclusive superordinate categories may also elicit different types of group representations. One important aspect may be related to how people perceive *citizens of the world* and *humans* in terms of their potential to emphasize similarities between the subgroups or to recognize and value both similarities and differences between subgroups.

In Study 2 participants were assigned to one of three experimental conditions: participants viewed a video presenting “what it means to identify” (1) as *citizens of the world*, (2) as *humans*, or (3) in a control condition, as *daughters and sons*. Then, using the same intergroup context as in Study 1, participants responded to the same measures of helping toward migrants, but only those assessing autonomy- and dependency-oriented help (we did not assess helping in general). Then, we assessed relative ingroup prototypicality of national citizens compared to migrants for the respective category, as well as the respective perceptions of essentialism, entitativity and group representations.

Considering previous research and the results of Study 1, we had two main hypotheses:

*H3*: We hypothesized that the salience of *citizens of the world* and *humans* would trigger higher dependency-oriented help, relative to the control category.

*H4*: We hypothesized that the salience of *humans* would trigger higher autonomy-oriented help, relative to the salience of *citizens of the world* and the control category.

Considering the unexpected relative outgroup prototypicality reported in Study 1, we also explored whether this finding would be replicated when manipulating the salience of *citizens of the world* and *humans*. Given the lack of previous research on the relationships of these specific all-inclusive identities with entitativity, essentialism, and group representations, we refrained from establishing directional hypotheses, acknowledging the exploratory nature of the study in this regard.

### Method

#### Participants and procedure

An *a priori* power analysis was conducted using G*Power (Faul et al., 2007). It indicated that a sample size of 246 participants would be required based on the predetermined parameters: effect size *f* = 0.20, power = 0.80, 3 groups, *α* = 0.05. Participants were recruited *via* Clickworker, in November and December 2020. We complied with APA Ethical Principles of Psychologists and Code of Conduct (APA-American Psychological Association, 2017), and the Code of Ethical Conduct in Research in place at the first author’s institution. All participants, who were 18 or older, completed an online survey in the Qualtrics platform in exchange for monetary compensation (~€3). All participants indicated informed consent and were debriefed. The validation procedures and inclusion criteria were the same used in Study 1. Participants who failed to respond correctly to questions about the experimental manipulation were also excluded. The informed consent was completed by 385 participants. However, 18 responses were excluded because they did not conform to the inclusion criterion about residency and citizenship in the host society. An additional 143 individuals were excluded because they failed validation procedures and/or questions about the manipulation. Thus, the final sample included 224 participants (slightly below the required sample size due to participants’ exclusion), who had sufficient proficiency in English, from 36 different countries (mostly United Kingdom, India, United States and South Africa; details in Supplementary material). The mean age of these participants was 35.32 years (*SD* = 11.51, range: 18–67), and 61.6% identified as a man and 37.1% as a woman (1.3% preferred not to answer); 75.9% had higher education; 58% were employed. Participants displayed heterogeneous political views (*M* = 3.75, *SD* = 1.50, range: 1–7, n = 223): 39.9% positioned themselves at the left/center-left; 34.5% at the center; and 25.6% at the right/center-right.

Participants were told that the survey aimed to understand how people use online platforms. After consenting to participate, participants were randomly assigned to one of three experimental conditions (i.e., *citizens of the world* vs. *humans* vs. control), watched a 2-min video containing the manipulations, and answered a few questions related to the video (used as exclusion criterion). The final sample was distributed per conditions as follows: *n_cit.world_* = 67; *n_human_* = 74; *n_control_* = 83.

Participants indicated their nationality and their own and their parents’ country of birth and residence. After the manipulation, the measures were administered in the following order^6^: group identification, helping preferences, helping orientations, relative ingroup prototypicality, entitativity, essentialism, perceptions of choice, evaluative status and valence, perceptions of group size, group representations, social dominance orientation, and national identification. Sociodemographic information was collected at the end, and participants were thanked and debriefed. The full protocol is available in Supplementary material.

#### Experimental manipulation

The experimental manipulation designed to vary the salience of different identities was introduced through a 2-min video, presenting the cover story. Participants were told that the first part of the survey investigated whether different online learning techniques (presentations with or without voice-over) help people retain information and that they would see a short video with content from an online psychology course. All participants were informed that they were assigned to a presentation without the voice-over. After the cover story, participants were presented with the manipulation: a PowerPoint presentation, entitled “Learning Psychology Online.” We selected “identification with groups” as the concept to be explained in the course. A description of what it means to identify with a large group was given (based on Leach et al., 2008), but the group used as an example varied across conditions. In one condition, participants read about identification with *citizens of the world*. In a second condition, the focus was identification with *humans*. In a third, control condition, the topic was identification with *daughters and sons*. We chose this as the control group because it also represents an all-inclusive category, that is, everyone is a daughter or son, but this identity is not related to the intergroup setting of migrants and host communities, representing thus a baseline for comparison. After watching the video, participants were asked four questions about its content (e.g., “Which example was given to exemplify the concept, in the presentation?), as a manipulation check. The verbatim instructions and manipulation check questions are available in Supplementary material.

### Materials

All items within each scale were randomized and were measured using a 7-point Likert-type scale (1 = *strongly disagree* to 7 = *strongly agree*), unless stated otherwise.

#### Group identification

Group identification was assessed to examine the preconditions for the occurrence of relative ingroup prototypicality, by a single item per target^7^: “I identify with [*citizens of the world* vs. *humans* vs. *daughters and sons*]” and “I identify with [national group]” (Postmes et al., 2013).

#### Helping preferences

Helping preferences were assessed as in Study 1, with slight adaptations. Instead of asking participants how they think a national user of the website should respond to a migrant’s request, we asked participants to select the solution they would themselves most likely adopt and recommend to future users of the website. As in Study 1, and confirmed by a multiple correspondence analysis,^8^ participants tended to display patterns of preferences for dependency- or autonomy-oriented help, independently of the scenario’s content. The measures were computed as in Study 1.

#### Helping orientations

Helping orientations for dependency-oriented help (5 items; *α* = 0.81) and for autonomy-oriented help (8 items; *α* = 0.90) were assessed as in Study 1.

#### Relative ingroup prototypicality

Relative ingroup prototypicality was measured by two separate items adapted from Waldzus et al. (2003), for ingroup prototypicality (“[National group] are prototypical [*citizens of the world* vs. *humans* vs. *daughters and sons*]”) and outgroup prototypicality (“Migrants are prototypical [*citizens of the world* vs. *humans* vs. *daughters and sons*]”). RIP was computed as in Study 1, that is, the difference between the mean scores of ingroup’ and outgroup’ prototypicality; positive scores indicate RIP; negative scores indicate relative outgroup prototypicality (ROP).

#### Evaluative status

Evaluative status of the categories is considered an important aspect to account for when examining group projection processes (Wenzel et al., 2016). As such, we included one additional item: “Generally speaking, people highly respect and admire *citizens of the world* vs. *humans* vs. *daughters and sons.*”

#### Valence

Valence of the categories, also an important aspect (Wenzel et al., 2016), was measured by the item “Generally speaking, people have a positive image of *citizens of the world* vs. *humans* vs. *daughters and sons.*”

#### Entitativity

Entitativity was assessed with 7 items measuring the extent to which the group was perceived as entitative (Lickel et al., 2000; Demoulin et al., 2006): groupness (1 = *not qualify at all as a group* to 7 = *very much qualify as a group*), members’ interaction (1 = *not interact at all with one another* to 7 = *interact very much with one another*), importance for its members (1 = *not at all important* to 7 = *very much important*), members’ common fate (1 = *not share a common fate* to 7 = *share a common fate*), members’ common goals (1 = *not have common goals* to 7 = *pursue common goals*), informativeness of belonging to the group (1 = *is not very informative* to 7 = *tells a lot about that person*) and similarity between members (1 = *diverse* to 7 = *similar*). Reliability scores for entitativity of each target category were not acceptable for all target categories, being very low for the social category *humans* (*α_cit.world_* = 0.75; *α_human_* = 0.47; *α_control_* = 0.81). Considering our goal of comparing entitativity between conditions we did not aggregate the items in a single index and will treat them separately in further analyses.

#### Perceptions of choice

Perceptions of choice over the group membership is an important aspect related to the mental representation of the group (Hamilton et al., 2004). As such, we included one item measuring the extent to which members have chosen to belong to the group (Toosi and Ambady, 2011; 1 *= is the result of a choice* to 7 = *does not result from a choice*; reverse coded).

#### Perceptions of group size

Perceptions of group size also an important aspect, was measured by the item “The group of *citizens of the world* vs. *humans* vs. *daughters and sons* includes every person on Earth.”

#### Essentialism

Essentialism was assessed by 5 items measuring the extent to which the group was perceived to be a natural-kind (Haslam et al., 2000; Demoulin et al., 2006): discreteness (1 = *clear-cut* to 7 = *fuzzy*; reverse coded), naturalness (1 = *artificial* to 7 = *natural*), immutability (1 = *easily changed* to 7 = *not easily changed*), stability (1 = *change much over time* to 7 = *change little over time*) and necessity (1 = *have necessary characteristics* to 7 = *do not have necessary characteristics*; reverse coded). Reliability scores for natural kindness dimensions (*α_cit.world_* = 0.42; *α_human_* = 0.51; *α_control_* = 0.53) were not acceptable for any of the social categories*.*^9^ For this reason, the indicators of essentialism are treated separately in subsequent analyses. Additionally, one item measured the attribution of essence to the group (underlying reality; Haslam et al., 2000; Demoulin et al., 2006; 1 = *has an underlying sameness* to 7 = *does not have an underlying sameness*, reverse coded).

#### Group representations

Group representations were assessed by 3 items, adapted from Guerra et al. (2015), measuring to what extent participants felt their national group and migrants’ group as a one-group (“When I think of migrants and [national group], who are living in [country of residence], I see them as one group”), as two subgroups of the same team (dual-identity) (“When I think of migrants and [nationality], who are living in [country of residence], I see them as two groups on the same team”) and as two separate groups (“When I think of migrants and [national group], who are living in [country of residence], I see them as two separate groups”).

#### Social dominance orientation

Social dominance orientation was assessed by 4 items of the Short SDO scale (Pratto et al., 2013; *α* = 0.70; e.g., “We should not push for group equality”).

### Results

#### Dependency- and autonomy-oriented help

To test H3 and H4, we examined the effects of condition (control, *citizens of the world*, or *humans*) on *preference for autonomy- relative to dependency-oriented help*, as well as on *orientations for dependency-oriented help* and *for autonomy-oriented help*.

As presented in Table 4, a one-way analysis of variance (ANOVA) examining differences between conditions did not reveal a significant effect of the manipulation on *preference for autonomy- relative to dependency-oriented help*. We explored differences between the conditions with simple contrasts: *citizens of the world* vs. *control*; *humans* vs. *control*; and *citizens of the world* vs. *humans*. None of the contrasts were significant, and only the comparison between *citizens of the world* vs. *humans* approached significance (*p* = 0.070), pointing to a tendency for participants in the condition making *humans* salient to score higher on *preference for autonomy- relative to dependency-oriented help.*^10^

**Table 4:** Means, *SD*s and differences regarding the impact of the categories “citizens of the world” and “humans.”

	Control (*n* = 83)	C. World (*n* = 67)	Humans (*n* = 74)	*Test*
	*M (SD)*	*M (SD)*	*M (SD)*	
**Helping preferences**				
Pr. for autonomy rel. to dependency	0.78 (0.23)	0.74 (0.23)	0.81 (0.22)	*F*(2, 221) = 1.666, *p* = 0.191, partial η^2^ = 0.01
**Helping orientations**				*F*(4, 440) = 0.662, *p* = 0.619; *Wilks’ Λ* = 0.988, partial η^2^ = 0.01
Or. for dependency	4.70 (1.01)	4.78 (1.16)	4.94 (1.08)	*F*(2, 221) = 0.986, *p* = 0.375
Or. for autonomy	5.83 (0.83)	5.82 (0.89)	5.98 (0.78)	*F*(2, 221) = 0.936, *p* = 0.394

A one-way multivariate analysis of variance (MANOVA) examining differences in *helping orientations* for *dependency-* and *autonomy-oriented help* between conditions^11^ (Table 4) did not reveal a significant multivariate effect of condition. Univariate effects and simple contrasts on each dependent variable were also not significant.

#### Relative ingroup prototypicality

First, to explore the occurrence of RIP,^12^ we analyzed whether the mean scores for relative ingroup prototypicality for the control group, *citizens of the world* or *humans* were significantly different from zero; then, we explored the mean differences for RIP between conditions.

As presented in Table 5, one sample *t*-tests revealed that means for RIP were not significantly different from zero for *citizens of the world* (*M* = 0.00, *SD* = 1.18, *t*[66] = 0.000, *p* = 1.00), *humans* (*M* = −0.12, *SD* = 0.66, t[73] = −1.583, *p* = 0.118) and the control group (*M* = 0.18, *SD* = 1.05, *t*[82] = −1.569, *p* = 0.120). That is, neither RIP nor ROP were observed in this study. Finally, a one-way ANOVA examining differences in relative ingroup prototypicality between conditions did not reveal a significant main effect of the experimental condition.

**Table 5:** Means, *SD*s and differences regarding the representation of the categories “citizens of the world” and “humans.”

	Control (*n* = 83)	Citiz. World (*n* = 67)	Humans (*n* = 74)	*Test*
	*M (SD)*	*M (SD)*	*M (SD)*	
**Relative ingroup prototypicality**	0.18 (1.05)	0.00 (1.18)	−0.12 (0.66)	*F*(2, 221) = 1.880, *p* = 0.155, partial η^2^ = 0.02
**Evaluative status**	4.87 (1.40)a,b	4.64 (1.38)a	5.26 (1.41)b	*F*(2, 221) = 3.519, ***p* = 0.031**
**Valence**	5.28 (1.23)a	4.73 (1.27)b	5.22 (1.27)a	*F*(2, 221) = 4.006, ***p* = 0.020**
**Entitativity**				*F*(14, 430) = 3.859, *p* < 0.001, *Wilks’ Λ* = 0.789, partial η^2^ = 0.11
Groupness	5.00 (1.64)a	4.81 (1.58)a	5.57 (1.51)b	*F*(2, 221) = 4.536, ***p* = 0.012**
Interaction	5.11 (1.35)a	4.82 (1.40)a	5.58 (1.22)b	*F*(2, 221) = 5.973, ***p* = 0.003**
Importance	4.80 (1.74)a	4.76 (1.72)a	5.61 (1.35)b	*F*(2, 221) = 6.512, ***p* = 0.002**
Common Fate	4.23 (1.88)	4.42 (1.73)	4.82 (1.57)	*F*(2, 221) = 2.354, *p* = 0.097
Common Goals	4.08 (1.73)a	4.69 (1.45)b	4.80 (1.42)b	*F*(2, 221) = 4.814, ***p* = 0.009**
Informativeness	3.42 (1.93)	3.97 (1.68)	3.65 (2.02)	*F*(2, 221) = 1.560, *p* = 0.212
Similarity	4.17 (1.89)	3.73 (1.70)	3.72 (1.68)	*F*(2, 221) = 1.665, *p* = 0.191
**Choice**	2.39 (1.55)a	3.27 (1.80)b	2.53 (1.71)a	*F*(2, 221) = 5.688, *p* = **0.004**
**Size**	5.64 (1.73)a	5.93 (1.31)a	6.62 (0.82)b	*F*(2, 221) = 10.657, *p <* **0.001**
**Natural kindness (Essentialism)**				*F*(10, 432) = 5.765, *p* < 0.001, *Wilks’ Λ* = 0.778, partial η^2^ = 0.12
Discreteness	5.04 (1.66)a	4.41 (1.74)b	5.23 (1.59)a	*F*(2, 220) = 4.618, *p* = **0.011**
Naturalness	6.01 (1.08)a	4.76 (1.61)b	5.74 (1.42)a	*F*(2, 220) = 16.565, *p <* **0.001**
Immutability	5.54 (1.57)a	4.77 (1.66)b	5.65 (1.47)a	*F*(2, 220) = 6.460, *p* = **0.002**
Stability	4.75 (1.80)a	4.38 (1.50)a,b	3.96 (1.63)b	*F*(2, 220) = 4.418, *p* = **0.013**
Necessity	4.49 (1.89)a	3.82 (1.72)b	4.97(1.65)a	*F*(2, 220) = 7.534, *p* **<** **0.001**
**Underlying reality (Essentialism)**	4.42 (1.72)a,b	4.21 (1.66)a	4.92 (1.59)b	*F*(2, 221) = 3.459, *p* **= 0.033**
**Group representations**				*F*(6, 438) = 1.465, *p* = 0.189, *Wilks’ Λ* = 0.961, partial η^2^ = 0.02
One-group	5.19 (1.93)	5.39 (1.91)	5.78 (1.75)	*F*(2, 221) = 2.015, *p* = 0.136
Dual identity	5.30 (1.77)a,b	5.19 (1.89)b	5.84 (1.95)a	*F*(2, 221) = 2.491, *p* = 0.085
Two separate groups	4.43 (2.03)	3.99 (2.13)	3.93 (2.16)	*F*(2, 221) = 1.350, *p* = 0.261

Different letters show significant differences between conditions as a result of pairwise comparisons (LSD).

#### Evaluative status

Additionally, we explored mean differences between conditions for categories’ evaluative status. A one-way ANOVA revealed a significant effect of salience on the evaluative status (Table 5). Pairwise comparisons showed that participants in the condition *humans* scored higher on respect and admiration relative to those in the *citizens of the world* condition.

#### Valence

We also explored mean differences between conditions for categories’ valence. A one-way ANOVA revealed a significant effect of salience on valence (Table 5). Pairwise comparisons showed that participants in the condition *citizens of the world* (vs. *humans*, and vs. control conditions) scored lower on the positive image toward the members of this group.

#### Entitativity

We explored mean differences between conditions for seven entitativity indicators (not assessed as a dimension). Results from a one-way MANOVA that considered the seven items as the dependent variables revealed a significant multivariate effect of condition on these indicators (Table 5). Follow-up one-way ANOVAs revealed a significant effect of condition on groupness, interaction, importance, and common goals; whereas there was not a significant effect on common fate, informativeness, and similarity (Table 5). Pairwise comparisons showed that participants in the *humans* condition, relative to those in the *citizens of the world* condition, scored higher on the perception that the category qualifies as a group (groupness), on the perceptions that members interact with one another (interaction), and on the importance of belonging to that group (importance). Additionally, participants in the control group (*daughters and sons*) scored significantly lower than did those in the condition of *humans* in terms of groupness, interaction, importance, and common goals; and they also scored lower than participants in the condition of *citizens of the world* in terms of common goals.

#### Perceptions of choice

We explored mean differences between conditions for perceptions of choice over the membership. A one-way ANOVA revealed a significant effect of condition on category’s choice (Table 5). Pairwise comparisons showed that participants in the condition of *citizens of the world*, relative to those in *humans* and control conditions, scored higher on the perception that membership in the category is the result of a choice.

#### Perceptions of group size

We explored mean differences between conditions for perceptions of group size. A one-way ANOVA revealed a significant effect of condition on category’s size (Table 5). Pairwise comparisons showed that participants in the condition of *humans* scored higher on the perception that the category includes everyone on Earth, relative to those in *citizens of the world* and control conditions.

#### Essentialism

We explored mean differences between conditions for six essentialism indicators (not assessed as a dimension), namely the five items that compose the natural kindness dimension, and the single item assessing underlying reality. Results from a one-way MANOVA, that considered the five items of natural kindness as the dependent variables, revealed a significant multivariate effect of condition on these indicators (Table 5). Follow-up one-way ANOVAs revealed a significant effect of salience on discreteness, naturalness, immutability, stability, and necessity (Table 5). Pairwise comparisons showed that participants in the *humans* condition, relative to those in the *citizens of the world* condition, scored higher on the perception that the category is clear-cut (discreteness), natural (naturalness), difficult to change (immutability) and its members are required to have necessary characteristics to justify the membership (necessity). The control condition (*daughters and sons*) was not different than *humans* in all aspects except for stability (higher mean), and differed significantly from *citizens of the world* in all aspects (higher means), except for stability. Regarding the essence of the group, a one-way ANOVA revealed a significant effect of the experimental condition on underlying reality (Table 5), and pairwise comparisons showed that participants in the *humans* condition scored higher on the perception that members of the category have similarities and differences on the surface but underneath they are basically the same, relative to those in the condition of *citizens of the world*.

#### Group representations

We explored mean differences between conditions for group representations. Results from a one-way MANOVA did not reveal a significant multivariate effect of condition on group representations (Table 5). Nonetheless, univariate effects showed a main effect of condition that approached significance (*p* = 0.085) for the dual-identity representation. Pairwise comparisons pointed to a tendency of participants in the condition of *humans* salience to score higher on dual-identity representations relative to those on the condition of *citizens of the world*.

### Discussion

Study 2 revealed, inconsistent with hypotheses, that when examining experimentally the potentially different impacts of making the all-inclusive superordinate categories *citizens of the world* and *humans* salient, no significant effects were observed on nationals’ inclination to offer dependency-oriented help (H2a) or for autonomy-oriented help (H2b) toward migrants. Moreover, the salience manipulation did not significantly influence patterns of relative ingroup nor outgroup prototypicality. Nonetheless, different patterns of how *citizens of the world* and *humans* are cognitively represented emerged. Overall, the category of *humans* triggered higher entitativity and essentialist beliefs compared to the category *citizens of the world*. The results regarding group representations pointed to a tendency for the salience of *humans* to activate a stronger representation of host community members and migrants as two subgroups of the same team (i.e., dual-identity representations), however, considering the lack of significant main effects, we should interpret these results with caution.

## General discussion

The main goal of the current research was to offer a new lens to better understand how different all-inclusive superordinate categories may produce different effects and to illuminate the psychological processes that account for different outcomes represented by responses to migrants. Considering *citizens of the world* and *humans*, commonly used all-inclusive categories, as labels for comparison, we examined their relationships to intergroup processes (Wenzel et al., 2016), cognitive representations (Gaertner et al., 2016), and impact on intergroup relations. These all-inclusive categories represent the highest level of identity abstraction (in categorical terms). We explored these relationships correlationally (Study 1) and experimentally (Study 2). To triangulate the similarities and differences between these all-inclusive categories, these studies also tested the effects of the strength identification with (Study 1) and the salience of (Study 2) these social categories, which are related but conceptually distinct constructs (Oakes et al., 1994; Ellemers et al., 1999; Wang and Dovidio, 2017). The process of identification with different all-inclusive categories might better predict intergroup outcomes given that it implies a stronger commitment to the specific group content (e.g., values, norms) than merely being exposed to information affecting the salience of these categories.

Overall, findings support the proposal that *citizens of the world* and *humans* differed in how they are cognitively represented with respect to relative ingroup prototypicality (albeit only in Study 1), as well as on perceptions of entitativity, essentialism, and to a lesser extent on the group representations (Study 2) they elicit for migrants. Regarding their impact on intergroup relations, the two studies did not converge, as only in Study 1 we found that level of identification with each of these categories was associated with different types of intergroup helping, whereas, in Study 2, the salience of these all-inclusive superordinate categories did not trigger different patterns of helping.

The overall findings show that the categories *citizens of the world* and *humans* differ in several structural aspects, and to a less extent on their impact suggesting that might be better represented as different socio-psychological realities. Our research was, admittedly, exploratory, and we acknowledge several limitations. However, these limitations also suggest promising directions for future research to more comprehensively understand the nature, correlates, and consequences of different all-inclusive categories.

### Relative ingroup prototypicality

Contrary to expectations, we did not observe relative ingroup prototypicality for *citizens of the world* or for *humans* in either of the current studies. Surprisingly, in Study 1 (but not in Study 2) relative outgroup prototypicality was observed for *citizens of the world*, as migrants were considered to be more prototypical for *citizens of the world* than participants’ national group. It is worth noting that different measures of relative ingroup prototypicality were used in each study.

These results are not in line with previous research revealing ingroup projection for all-inclusive superordinate categories (e.g., Bilewicz and Bilewicz, 2012, “humanity”; Paladino and Vaes, 2009, “human”; Reese et al., 2012, 2016, “world population”). We can only speculate about possible explanations regarding the occurrence of relative outgroup prototypicality for *citizens of the world* in Study 1. Wenzel et al. (2016) proposed two aspects of how superordinate categories are represented that might equate the perceptions of prototypicality between groups: (a) the vagueness of the superordinate categories so that no subgroup can claim to better represent the undefined prototype; (b) the diversity (i.e., intra-category differences) of the superordinate categories so that different subgroups can be equally prototypical. Previous research has shown that people can differentiate specific attributes to both *citizens of the world* and *humans* (e.g., Carmona et al., 2020), indicating that these are not generally perceived to be vague and undefinable categories. Therefore, it seems plausible to discard the argument of vagueness to explain the absence of a relative ingroup prototypicality effect. As such, we advance alternative explanations. *Citizens of the world* may be seen as a particularly diverse category such that one’s own group may be viewed as equally prototypical of this category, or even less prototypical than people, like *migrants*, who were formerly *citizens* of many different countries. Importantly, one explanation for one’s group to be seen as less prototypical may be related to the potentially malleable prototypical meaning of *citizens of the world*. In certain circumstances people use this label to describe those who move around the world, who interact with different cultures and seem to have “no roots” nor a special bond to their country of origin (Türken and Rudmin, 2013; Carmona et al., 2022). Considering this meaning, the prototype of *migrants* (as those who live outside their country of origin) may be seen as more similar to the prototype of *citizens of the world*, than the one of one’s own national group, which may therefore be seen as less prototypical for this superordinate category.

Even so, our results for the category of *humans* appear inconsistent with previous research. However, seemingly minor difference in phrasing may have fairly strong and systematic impact. Specifically, the terms used in previous research may have emphasized similarity/homogeneity—humanity, all humans, and human (implying a particular quality)—more than the term humans, which might allow for the category to be perceived as more diverse/heterogeneous in which group members differ greatly from one another and do not share many characteristics. The recognition, and perhaps acceptance, of differences between *humans* might have led to the perception of equal prototypicality between the national group and migrants. Thus, future research on relative ingroup prototypicality associated with all-inclusive categories might also assess perceptions of the complexity of these social categories directly, as well as consider how differences in phrasing that may emphasize either similarity or difference can affect group projection processes and, ultimately, other outcomes.

### Group representations

The value of studying in future research the degree to which all-inclusive categories emphasize similarities versus differences may also further illuminate the pattern of findings we observed for group representations. A possible explanation for the finding that *humans* may tend to elicit a stronger dual-identity representation than *citizens of the world* might be that the category *humans* could be more effective in simultaneously emphasizing both similarities and differences among people (e.g., “all different, all equal,” most likely in biological aspects). Also, participants perceived greater interaction between *humans* than between *citizens of the world*, which is a factor that can elicit the recategorization of two subgroups into a superordinate aggregate, either by one-group or dual-identity representations (Gaertner et al., 2016).

### Entitativity and essentialism

Our results suggested that all-inclusive superordinate categories that encompass everyone, such as *citizens of the world* and *humans*, can be perceived as a group, in common sense, complying sufficiently with most requirements for entitativity. Nonetheless, the category *humans* scored significantly higher on several indicators of entitativity and essentialist beliefs, suggesting that people more strongly perceive the aggregate of *humans* (vs. *citizens of the world*) as a group, in which members are bonded together by an underlying essence. These results are in line with previous research showing that *humans* tend to be essentialized and perceived as having a biologically based essence (Haslam et al., 2005). One possible explanation might be related to the spontaneous meanings that people themselves attribute to these categories. Previous research suggested that humanness-oriented labels (e.g., *all humans everywhere*) might activate more biologically based attributes (e.g., physical, emotional attributes), whereas global citizenship-oriented labels (e.g., *citizens of the world*) might activate more attitudinal based attributes (e.g., multiculturalism; cosmopolitanism; Carmona et al., 2020). Thus, we suggest that the biological-based content activated by humanness-oriented labels might boost essentialist beliefs about human nature, which is in line with research on humanness essence (e.g., Haslam et al., 2005). This is important considering that essentialist beliefs have been associated with negative effects of appealing to common humanity (e.g., Greenaway et al., 2011; Morton and Postmes, 2011). We note that these meanings might reflect the worldviews of the western socio-cultural context in which the research was carried out. Considering the potential cross-cultural variability, further research is needed to replicate these findings in different cultural contexts.

### Intergroup helping

Contrary to expectations, identification with *citizens of the world* and *humans* were associated with different types of helping responses but manipulating the salience of these categories did not trigger different patterns of helping.

A possible explanation for the association between identification with *citizens of the world* and the tendency to offer dependency-oriented help or opposition to help (beyond the measurement issue) could be related to the different prototypical contents activated by this category. That is, the prototypical content of the category *citizens of the world* could have been experienced as a threat and triggered defensive helping (i.e., dependency-oriented help) or opposition to help. That is, the idea of what it means to be a *citizen of the world* seems to be related to multicultural and cosmopolitan views, which might reflect a worldview influenced by a globalized Western culture and might be malleable to contextual socio-status-political motives (Rosenmann et al., 2016). It is therefore plausible that when thinking about how much they identify themselves with *citizens of the world*, individuals might activate a prototype mostly composed of the attitudinal aspects that people share as members of a global political community, such as the endorsement of multiculturalism and cosmopolitanism. If that was the case, the identification with *citizens of the world* might have activated existing political divisions in society regarding multiculturalist views and could have been experienced as a threat by some host communities’ members, particularly considering that national identification was also salient in this context. If so, the tendency to offer dependency-oriented help or opposition to help, might be linked to the motivation of host community members to maintain the status quo, namely their advantageous social position and their role as providers of help.

An alternative explanation for the association between identification with *humans* and the tendency to offer multiple types of help could be that when thinking about how much they identify with *humans*, individuals might have activated a category prototype mostly composed of the biologically based aspects that people share as members of the human species (e.g., physical appearance, need of bonding). If that was the case, identification with *humans* might have been less malleable to contextual socio-status-political motives, which might have been experienced as less threatening.

### General limitations

In addition to the specific limitations and suggestions for future research, we acknowledge some general methodological limitations of the current work, which speak to conceptual issues to consider in future research.

One important aspect to consider is that in the current study participants were conceptualized as members of an advantaged group in numerical, economic, and social terms. Previous research has shown that different inclusive representations are preferred and have different consequences for groups of unequal status, depending on the cultural and historical context, or the groups’ goals (e.g., Hehman et al., 2012). Whereas some research suggests that advantaged groups favor more assimilationist orientations, such as one-group representations (e.g., Dovidio et al., 2001), other research shows that they also endorse dual-identity representations, as these might mitigate threats to the ingroup distinctiveness and higher status within the superordinate category (Guerra et al., 2010, 2013; Gaertner et al., 2016). Thus, it is important that future research explores how advantaged and disadvantaged groups conceive of all-inclusive superordinate categories, as well as the potential role of distinctiveness motivations. Additional research might also investigate how personal and contextual influences shape the impact of all-inclusive identities. For example, as showed by Hackett and Hogg (2014), experiencing greater self-uncertainty tended to undermine community identification when diversity was important. Thus, future work might consider, along with the specific nature of all-inclusive identities, the role of personal and contextual factors.

One strength of these studies is that they cover a broad range of national samples. However, we caution that while there may be basic similarities in the ingroup-outgroup dynamics with respect to migrants across national contexts, there is also likely that how people think about migrants may vary as a function of geographical, historical, economic, and political influences. These cultural differences in the conceptualization of migrants, which we did not assess, could have also impacted our findings, and deserve to be investigated in future research (see, for example, Esses et al., 2006).

We highlight four additional directions for future research. First, that the category labels used for comparison might drive different connotations and meanings in different cultures and languages (McFarland, 2017). Considering that our studies were conducted with international samples using the English language, they are not sensitive to translation and interpretation issues that might have occurred. As such, we recommend further research to examine the meanings of different labels and their intergroup outcomes, in cross-national samples and languages. Second, different measures were used in both studies to assess the same construct (e.g., RIP), due to concerns about the length of the studies. However, we cannot rule out that this might partially account for the lack of replication of some findings. Relatedly, the lack of replication might be due to the different designs employed in each study, which is not a limitation but an important aspect to consider. Third, our measure of *helping preferences* was designed for the present study, and it was not previously validated. Fourth, considering the lack of previous studies analyzing the relations between our main variables, the current studies are exploratory in nature. Thus, it is important that future research replicates and tests directional hypotheses, as well as uses other settings than online platforms (e.g., laboratory and real groups), and other target groups.

## Conclusion

Understanding the effects of all-inclusive social categories on how people think about, feel about, and behave toward migrants, specifically, and people originally conceived of as outgroup members is valuable both theoretically and practically.

One particularly key finding is that the effects of all-inclusive identities can differ in important ways as a function of the specific identity involved. Overall, the current studies suggest that the all-inclusive superordinate categories *citizens of the world* and *humans* might be better represented as different socio-psychological realities, given their differences in terms of *structure* and *impact*. In light of these findings and interpretations, we corroborate the proposition that the interchangeable use of different labels is problematic, considering these might activate different content and thus different identity and intergroup processes, as well as behavioral consequences (Reysen and Katzarska-Miller, 2015; Reese et al., 2016), which could partly account for the inconsistencies in their intergroup outcomes.

Beyond the theoretical contribution, we expect the current work to provide researchers, policymakers, educators, or practitioners an awareness about the need to critically account for the complexity of appealing to all-inclusive forms of identification and considering their meanings within the structural systems of power in which they are used. In fact, one approach to mobilize people to take prosocial actions on global matters has been to enhance a sense of togetherness, by using statements such as “we are all citizens of the world” or “we are all humans.” Our concern is that, in a polarized world, the salience of different all-inclusive superordinate categories might drive undesirable societal outcomes, such as the maintenance of the status quo between groups of unequal status. To our understanding, one of the core questions in terms of impact is not simply whether all-inclusive identities promote prosocial behavior - there is evidence that they generally do – but whether they promote empowering interactions, capable of reducing or eliminating the social disparity, and promote social change. Further research is needed to continue the search for the optimal conditions under which all-inclusive superordinate categories might contribute to solve urgent global issues, building more inclusive societies, and ultimately foster socio-structural equality worldwide.

## Data availability statement

The raw data supporting the conclusions of this article will be made available by the authors, without undue reservation.

## Ethics statement

Ethical review and approval were not required for the study on human participants in accordance with the local legislation and institutional requirements. The patients/participants provided their written informed consent to participate in this study.

## Author contributions

MC conceptualized and developed the design of the studies, under the supervision of RG and JH, as well as in collaboration with JD and DS. MC conducted the data collection, organized the database, performed the statistical analysis and wrote the first draft of the manuscript. All authors wrote/edited sections of the present manuscript and contributed to manuscript critical review and approved the submitted version.

## Funding

This work was funded by Fundação para a Ciência e Tecnologia (FCT, Portugal), with a grant/PhD studentship awarded to the first author (PD/BD/129601/2017), as well as supported, in part, by NSF Grant #1823763 awarded to the third author.

## Conflict of interest

The authors declare that the research was conducted in the absence of any commercial or financial relationships that could be construed as a potential conflict of interest.

## Publisher’s note

All claims expressed in this article are solely those of the authors and do not necessarily represent those of their affiliated organizations, or those of the publisher, the editors and the reviewers. Any product that may be evaluated in this article, or claim that may be made by its manufacturer, is not guaranteed or endorsed by the publisher.

## Footnotes

^1^One additional measure was administered but not analysed.

^2^Details in Supplementary material.

^3^Details in Supplementary material.

^4^Three from the 8 items were removed by EFA (see Supplementary material).

^5^The preconditions for RIP were satisfied (see Supplementary material).

^6^Additional measures were administrated but not included in the analysis.

^7^Two subdimensions of the MIIS were administered but not analysed.

^8^Details in Supplementary material.

^9^EFAs were run for each condition, however none of the final solutions reproduced the theoretical dimensions for the social categories under analysis.

^10^When political orientation was included as a covariate, results for the ANCOVA and simple contrasts were identical. When social dominance orientation was included as a covariate, the marginal effect on simple contrasts was not observed.

^11^When political orientation and social dominance orientation were included as covariates, results for the MANCOVA’s omnibus tests and simple contrasts were identical.

^12^The preconditions for RIP were satisfied (see Supplementary material).
