# Supplementary material for: Unpacking all-inclusive superordinate categories: Comparing correlates and consequences of global citizenship and human identities
Source: Front Psychol. 2022 Sep 2;13:986075. doi: 10.3389/fpsyg.2022.986075 (PMC9491308; doi:10.3389/fpsyg.2022.986075)
Supplement: Supplementary file 1 [file Data_Sheet_1.pdf]

Supplementary Material

This document contains a detailed description of the materials used and additional analysis.

1 Study 1 3

1.1 Full protocol 3

1.1.1 Informed consent 3

1.1.2 Eligibility: Country of birth, residence and nationality 4

1.1.3 Group identification 8

1.1.4 Altruistic orientation 9

1.1.5 Perceived diversity of the superordinate categories 11

1.1.6 Relative ingroup prototypicality 11

1.1.7 Helping preferences 12

1.1.8 Helping orientations 16

1.1.9 Sociodemographic information 17

1.1.10 Debriefing 18

1.2 Additional analysis 19

1.2.1 Additional information about participants in Study 1 19

1.2.2 Group identification: Additional analysis of the measure used in Study 1 19

1.2.3 Altruistic orientation: Additional analysis of the measure used in Study 1 20

1.2.4 Perceived diversity of superordinate categories: Secondary analysis of Study 1 21

1.2.5 RIP: Additional analysis of the measure used in Study 1 21

1.2.6 Helping preferences: Additional analysis of the measure used in Study 1 22

1.2.7 Helping orientations: Additional analysis of the measure used in Study 1 24

1.2.8 Means, SDs, and zero-order correlations among all variables of Study 1 26

1.2.9 RIP: Examining the preconditions for ingroup projection in Study 1 27

1.2.10 Predicting helping preferences by type in Study 1 27

2 Study 2 28

2.1 Full protocol 28

2.1.1 Informed consent 28

2.1.2 Eligibility: Nationality and country of residence 29

2.1.3 Experimental manipulation 29

2.1.4 Manipulation check 31

2.1.5 Group identification 31

2.1.6 Helping preferences 32

2.1.7 Willingness to help 34

2.1.8 Costs and benefits of helping 34

2.1.9 Helping orientations 35

2.1.10 Willingness to participate in collective action 35

2.1.11 Feelings towards migrants 35

2.1.12 Relative prototypicality 35

2.1.13 Entitativity 36

2.1.14 Essentialism 37

2.1.15 Perceptions of choice 38

2.1.16 Evaluative status and valence 38
2.1.17 Perceptions of group size 38
2.1.18 Group representations 38
2.1.19 Migrant’s origin 39
2.1.20 Social dominance orientation 39
2.1.21 National identification 39
2.1.22 Sociodemographic information 40
2.1.23 Debriefing/ 41
2.2 Additional analysis 42
2.2.1 Additional information about participants in Study 2 42
2.2.2 Helping preferences: Additional analysis of the measure used in Study 2 42
2.2.3 RIP: Examining the preconditions for ingroup projection in Study 2 44
2.2.4 Collective action and feeling towards migrants: Secondary analysis of Study 2 44

1 Study 1

1.1 Full protocol

Study 1: List and order of measures

- Informed consent
- Eligibility: Country of birth, residence and nationality
- Group identification
- Social value orientation
- Perceived diversity of "citizens of the world" and "humans"
- Relative ingroup prototypicality
- Helping preferences
- Helping orientations
- Sociodemographic information
- Debriefing / Completion code

1.1.1 Informed consent

Welcome to our study!

ISCTE-University Institute of Lisbon (Portugal) is studying what people think about migration experiences. To get the most out of this survey, we ask you to fill out all the questions. In total, this should take approximately 20 minutes.

The information that you provide will not be used to judge you in any way, and this research follows the recommendations of the CIS-IUL Ethics Committee. We are interested in the first answer that comes to your mind, so there are no right or wrong answers

If you want more information, now or in the future, you are free to contact the researchers by e-mail (dr. Margarida Carmona - mgfcl@iscte-iul.pt). Thank you! The research team,

Please read the following consent: I am aged 18 years or older. I agree to voluntarily participate in this study. I am free to withdraw at any time, without giving a reason. If my results are used in scientific publications, or are published in any other way, my data will be completely anonymous. My data will not be sent to third parties. Only researchers will have access to data. There are no physical, legal or economic risks associated with participating in this study.

At the end of the survey, you will be given a unique completion code to insert in MTurk platform. Be sure to enter your completion code correctly to ensure prompt payment.

I confirm that I have read and understood the above and freely consent to participating in this study:

Yes / No

1.1.2 Eligibility: Country of birth, residence and nationality

CAPTCHA Test (Robot check)

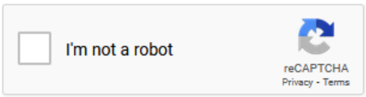

Country of birth

____

Country of residence at the moment (Country order from A to Z)

- Afghanistan
- Albania
- Algeria
- Andorra
- Angola
- Antigua and Barbuda
- Argentina
- Armenia
- Australia
- Austria
- Azerbaijan
- Bahamas
- Bahrain
- Bangladesh
- Barbados
- Belarus
- Belgium
- Belize
- Benin
- Bhutan
- Bolivia
- Bosnia and Herzegovina
- Botswana
- Brazil
- Brunei
- Bulgaria
- Burkina Faso
- Burundi
- Cape Verde
- Cambodia
- Cameroon
- Canada
- Central African Republic
- Chad
- Chile
- China
- Colombia
- Comoros
- Congo, Democratic Republic of the
- Congo, Republic of the
- Costa Rica
- Cote d'Ivoire
- Croatia
- Cuba
- Cyprus
- Czech Republic
- Denmark
- Djibouti
- Dominica
- Dominican Republic
- Ecuador
- Egypt
- El Salvador
- Equatorial Guinea
- Eritrea
- Estonia
- Eswatini
- Ethiopia
- Fiji
- Finland
- France
- Gabon
- Gambia
- Georgia
- Germany
- Ghana
- Greece
- Grenada
- Guatemala
- Guinea
- Guinea-Bissau
- Guyana
- Haiti
- Honduras
- Hungary
- Iceland
- India
- Indonesia
- Iran
- Iraq
- Ireland
- Israel
- Italy
- Jamaica
- Japan
- Jordan
- Kazakhstan
- Kenya
- Kiribati
- Kosovo
- Kuwait
- Kyrgyzstan
- Laos
- Latvia
- Lebanon
- Lesotho
- Liberia
- Libya

- Liechtenstein
- Lithuania
- Luxembourg
- Madagascar
- Malawi
- Malaysia
- Maldives
- Mali
- Malta
- Marshall Islands
- Mauritania
- Mauritius
- Mexico
- Micronesia
- Moldova
- Monaco
- Mongolia
- Montenegro
- Morocco
- Mozambique
- Myanmar
- Namibia
- Nauru
- Nepal
- Netherlands
- New Zealand
- Nicaragua
- Niger
- Nigeria
- North Korea
- North Macedonia
- Norway
- Oman
- Pakistan
- Palau
- Palestine
- Panama
- Papua New Guinea
- Paraguay
- Peru
- Philippines
- Poland
- Portugal
- Qatar
- Romania
- Russia
- Rwanda
- Saint Kitts and Nevis
- Saint Lucia
- Saint Vincent and the Grenadines
- Samoa
- San Marino
- Sao Tome and Principe
- Saudi Arabia
- Senegal
- Serbia
- Seychelles
- Sierra Leone
- Singapore
- Slovakia
- Slovenia
- Solomon Islands
- Somalia
- South Africa
- South Korea
- South Sudan
- Spain
- Sri Lanka
- Sudan
- Suriname
- Sweden
- Switzerland
- Syria
- Taiwan
- Tajikistan
- Tanzania
- Thailand
- Timor-Leste
- Togo
- Tonga
- Trinidad and Tobago
- Tunisia
- Turkey
- Turkmenistan
- Tuvalu
- Uganda
- Ukraine
- United Arab Emirates
- United Kingdom
- United States of America
- Uruguay
- Uzbekistan
- Vanuatu
- Vatican City (Holy See)
- Venezuela
- Vietnam
- Yemen
- Zambia
- Zimbabwe

Nationality (Country order from A to Z). If you are a dual-citizen, pick one nationality to select here, and then write down the second one below.

- Afghan
- Albanian
- Algerian
- Andorran
- Angolan
- Antiguan/Barbudan
- Argentine
- Armenian
- Australian
- Austrian
- Azerbaijani
- Bahamian
- Bahraini
- Bangladeshi
- Barbadian
- Belarusian
- Belgian
- Belizean
- Beninese
- Bhutanese
- Bolivian

- Bosnian/Herzegovinian
- Batswana
- Brazilian
- Bruneian
- Bulgarian
- Burkinabe
- Burundian
- Cape Verdean
- Cambodian
- Cameroonian
- Canadian
- Central African
- Chadian
- Chilean
- Chinese
- Colombian
- Comoran
- Congolese (DRC)
- Congolese (RC)
- Costa Rican
- Ivoirian
- Croatian
- Cuban
- Cypriot
- Czech
- Danish
- Djiboutian
- Dominican
- Dominican (DR)
- Ecuador
- Egyptian
- Salvadoran
- Equatorial Guinean
- Eritrean
- Estonian
- Swazi
- Ethiopian
- Fijian
- Finnish
- French
- Gabonese
- Gambian
- Georgian
- German
- Ghanaian
- Greek
- Grenadian
- Guatemalan
- Guinean
- Bissau-Guinean
- Guyanese
- Haitian
- Honduran
- Hungarian
- Icelandic
- Indian
- Indonesian
- Iranian
- Iraqi
- Irish
- Israeli
- Italian
- Jamaican
- Japanese
- Jordanian
- Kazakhstani
- Kenyan
- I-Kiribati
- Kosovan
- Kuwaiti
- Kyrgyzstani
- Laotian
- Latvian
- Lebanese
- Basotho
- Liberian
- Libyan
- Liechtenstein
- Lithuanian
- Luxembourg
- Malagasy
- Malawian
- Malaysian
- Maldivian
- Malian
- Maltese
- Marshallese
- Mauritanian
- Mauritian
- Mexican
- Micronesian
- Moldovan
- Monegasque
- Mongolian
- Montenegrin
- Moroccan
- Mozambican
- Myanmarese
- Namibian
- Nauruan
- Nepali
- Dutch
- New Zealand
- Nicaraguan
- Nigerien
- Nigerian
- North Korean
- North Macedonian
- Norwegian
- Omani
- Pakistani
- Palauan
- Palestinian
- Panamanian
- Papua New Guinean
- Paraguayan
- Peruvian
- Philippine
- Polish
- Portuguese
- Qatari
- Romanian
- Russian
- Rwandan
- Kittitian/Nevisian
- Saint Lucian
- Saint Vincentian
- Samoan
- Sammarinese
- Sao Tomean
- Saudi
- Senegalese

- Serbian
- Seychellois
- Sierra Leonean
- Singaporean
- Slovak
- Slovenian
- Solomon Islander
- Somali
- South African
- South Korean
- South Sudanese
- Spanish
- Sri Lankan
- Sudanese
- Surinamese
- Swedish
- Swiss
- Syrian
- Taiwanese
- Tajikistani
- Tanzanian
- Thai
- Timorese
- Togolese
- Tongan
- Trinidadian/Tobagonian
- Tunisia
- Turkey
- Turkmen
- Tuvaluan
- Ugandan
- Ukrainian
- UA-Emirati
- British
- US-American
- Uruguayan
- Uzbekistani
- Ni-Vanuatu
- Vatican
- Venezuelan
- Vietnamese
- Yemeni
- Zambian
- Zimbabwean

If you are a dual-citizen, please indicate your second nationality.
____

1.1.3 Group identification

Multicomponent Ingroup Identification Scale by Leach et al. (2008)

Please indicate how much you agree or disagree with the following statements, using the scale.
1 Strongly disagree
2 Disagree
3 Somewhat disagree
4 Neither agree nor disagree
5 Somewhat agree
6 Agree
7 Strongly agree

Global citizenship identification (14 items)

- I think that citizens of the world have a lot to be proud of.
- It is pleasant to be a citizen of the world.
- Being a citizen of the world gives me a good feeling.
- I am glad to be a citizen of the world.
- I often think about the fact that I am a citizen of the world.
- The fact that I am a citizen of the world is an important part of my identity.
- Being a citizen of the world is an important part of how I see myself.
- I feel a bond with citizens of the world.
- I feel solidarity with citizens of the world.
- I feel committed to citizens of the world.
- I have a lot in common with the average citizen of the world.
- I am similar to the average citizen of the world.
- Citizens of the world have a lot in common with each other.
- Citizens of the world are very similar to each other.
- *This is a control question to screen out random clicking. Please select "disagree" to demonstrate you have read this.*

Human identification (14 items)

- I think that humans have a lot to be proud of.
- It is pleasant to be a human.
- Being a human gives me a good feeling.
- I am glad to be a human.
- I often think about the fact that I am a human.
- The fact that I am a human is an important part of my identity.
- Being a human is an important part of how I see myself.
- I feel a bond with humans.
- I feel solidarity with humans.
- I feel committed to humans.
- I have a lot in common with the average human.

- I am similar to the average human.
- Humans have a lot in common with each other.
- Humans are very similar to each other.
- *This is a control question to screen out random clicking. Please select "disagree" to demonstrate you have read this.*

National identification (14 items)

- I think that the [national group] have a lot to be proud of.
- It is pleasant to be [national group].
- Being [national group] gives me a good feeling.
- I am glad to be [national group].
- I often think about the fact that I am [national group].
- The fact that I am [national group] is an important part of my identity.
- Being [national group] is an important part of how I see myself.
- I feel a bond with the [national group].
- I feel solidarity with the [national group].
- I feel committed to the [national group].
- I have a lot in common with the average [national group].
- I am similar to the average [national group].
- The [national group] have a lot in common with each other.
- The [national group] are very similar to each other.
- *This is a control question to screen out random clicking. Please select "disagree" to demonstrate you have read this.*

1.1.4 Altruistic orientation

Social Value Orientation (SVO) Slider Measure by Murphy et al. (2011)

Instructions

In this task, imagine that you have been randomly paired with another person, whom we will refer to as the other. This other person is someone you do not know and will remain mutually anonymous. All of your choices would be completely confidential. You will be making a series of decisions about allocating resources between you and this other person. For each of the following questions, please indicate the distribution you prefer most by selecting the button below the payoff allocations (points that can be converted into real money). You can only make one selection for each question. Your decisions will yield money for both yourself and the other person. In the example below, a person has chosen to distribute the payoff so that he/she receives 50 points, while the anonymous other person receives 40 points.

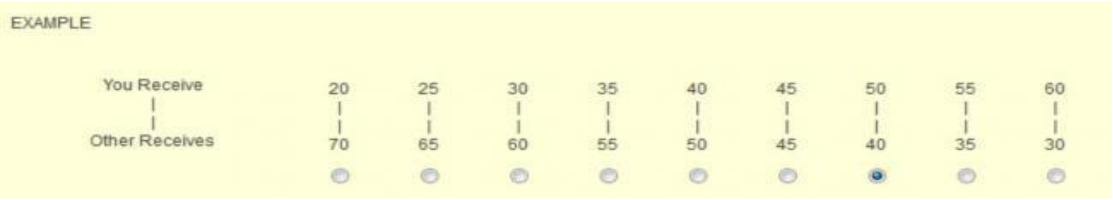

There are no right or wrong answers, this is all about personal preferences. After you have made your decision, select the resulting distribution of points by clicking on button below your

choice. As you can see, your choices will influence both the amount of money you receive as well as the amount of money the other receives

Item 1

You Receive	85	85	85	85	85	85	85	85	85
Other Receives	85	76	68	59	50	41	33	24	15

Item 2

You Receive	85	87	89	91	93	94	96	98	100
Other Receives	15	19	24	28	33	37	41	46	50

Item 3

You Receive	50	54	59	63	68	72	76	81	85
Other Receives	100	98	96	94	93	91	89	87	85

Item 4

You Receive	50	54	59	63	68	72	76	81	85
Other Receives	100	89	79	68	58	47	36	26	15

Item 5

You Receive	100	94	88	81	75	69	63	56	50
Other Receives	50	56	63	69	75	81	88	94	100

Item 6

You Receive	100	98	96	94	93	91	89	87	85
Other Receives	50	54	59	63	68	72	76	81	85
	○	○	○	○	○	○	○	○	○

1.1.5 Perceived diversity of the superordinate categories

Adapted from Waldzus et al., 2003

Please indicate how much you agree or disagree with the following statements, using the scale.
1 Strongly disagree
2 Disagree
3 Somewhat disagree
4 Neither agree nor disagree
5 Somewhat agree
6 Agree
7 Strongly agree

Perceived diversity of "citizens of the world" (4 items)

- There is not "the one" typical citizen of the world but rather many different kinds of citizens of the world.
- One of citizen of the world's characteristics is its great diversity.
- Citizens of the world share a lot of common attributes.
- Citizens of the world similarities outweigh their differences.

Perceived diversity of "humans" (4 items)

- There is not "the one" typical human but rather many different kinds of humans.
- One of humans's characteristics is its great diversity.
- Humans share a lot of common attributes.
- Humans similarities outweigh their differences.

1.1.6 Relative ingroup prototypicality

Adapted from Wenzel et al. (2003)

Ingroup attributes
Please write down 3 attributes that you consider characteristic of [national group], compared to migrants:
1. __*Ingroup attribute 1*__
2. __*Ingroup attribute 2*__
3. __*Ingroup attribute 3*__

Outgroup attributes
Please write down 3 attributes that you consider characteristic of migrants, compared to [national group]
1. __*Outgroup attribute 1*__
2. __*Outgroup attribute 2*__
3. __*Outgroup attribute 3*__

When answering these questions, you thought of migrants living in:
[List of countries]

Relative ingroup prototypicality for "citizens of the world"
Please, rate to what extent each attribute you've mentioned applies to CITIZENS OF THE WORLD.

1 Does not apply at all to CITIZENS OF THE WORLD
7 Applies very much to CITIZENS OF THE WORLD

1. __*Ingroup attribute 1*__
2. __*Ingroup attribute 2*__
3. __*Ingroup attribute 3*__
4. __*Outgroup attribute 1*__
5. __*Outgroup attribute 2*__
6. __*Outgroup attribute 3*__

Relative ingroup prototypicality for "humans"
Please, rate to what extent each attribute you've mentioned applies to HUMANS.

1 Does not apply at all to HUMANS
7 Applies very much to HUMANS

1. __*Ingroup attribute 1*__
2. __*Ingroup attribute 2*__
3. __*Ingroup attribute 3*__
4. __*Outgroup attribute 1*__
5. __*Outgroup attribute 2*__
6. __*Outgroup attribute 3*__

1.1.7 Helping preferences

Adapted from Halabi et al. (2008)

Instructions
Please watch carefully this 30 second video (press Play button to start. If you are using a mobile phone, please click on expansion screen button).

Introductory video' shots:

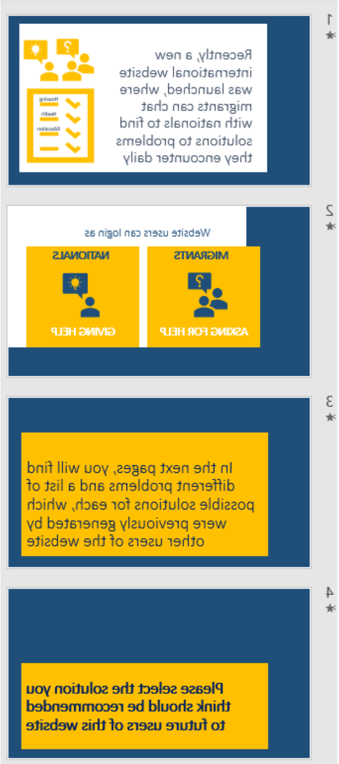

Scenarios

Scenario 1
Problem presented by a migrant user:
"I need to make an appointment in a health facility"
As [nationality], please select the solution you think should be recommended to future users of the website:

- ***Dependency:*** The [nationality] user should contact the health facility and make the appointment for the migrant user
- ***Autonomy:*** The [nationality] user should inform and support the migrant user on how to identify a health facility and how to make an appointment
- ***No help:*** The [nationality] user shouldn't help, because the migrant user should find a solution to this problem on his/her own"
- None of the above.

Scenario 2
Problem presented by a migrant user: "I have an appointment with the school teacher of my children, but we don't speak a common language "
As [nationality], please select the solution you think should be recommended to future users of the website:

- ***Dependency:*** The [nationality] user should find an official interpreter to be present in the meeting
- ***Autonomy:*** The [nationality] user should inform and support the migrant user on how to find an official interpreter to be present at the meeting
- ***No help:*** The [nationality] user shouldn't help, because the migrant user should find a solution to this problem on his/her own"
- None of the above.

Scenario 3
Problem presented by a migrant user: “I would like to bring my family to the new country I’m living in "
As [nationality], please select the solution you think should be recommended to future users of the website:

- ***Dependency:*** The [nationality] user should contact the appropriate government services to get information about the legal procedures
- ***Autonomy:*** The [nationality] user should inform and support the migrant user regarding the appropriate government services to get information about the legal procedures
- ***No help:*** The [nationality] user shouldn’t help, because the migrant user should find a solution to this problem on his/her own”
- None of the above.

Scenario 4
Problem presented by a migrant user: "I was a victim of discrimination."
As [nationality], please select the solution you think should be recommended to future users of the website:

- ***Dependency:*** The [nationality] user should report the incident to the legal authorities
- ***Autonomy:*** The [nationality] user should inform and support the migrant user on how to report the incident to the legal authorities
- ***No help:*** The [nationality] user shouldn’t help, because the migrant user should find a solution to this problem on his/her own”
- None of the above.

Scenario 5
Problem presented by a migrant user: " I need to create a CV to apply to job in the new country."
As [nationality], please select the solution you think should be recommended to future users of the website:

- ***Dependency:*** The [nationality] user should prepare the CV for the migrant user
- ***Autonomy:*** The [nationality] user should inform and support the migrant user on how to prepare a good CV
- ***No help:*** The [nationality] user shouldn’t help, because the migrant user should find a solution to this problem on his/her own”
- None of the above.

Scenario 6
Problem presented by a migrant user: "I have a tourist visa, but I want to obtain a residence permit”
As [nationality], please select the solution you think should be recommended to future users of the website:

- ***Dependency***: The [nationality] user should contact the appropriate government services to get information about the legal procedures
- ***Autonomy***: The [nationality] user should inform and support the migrant user regarding the appropriate government services to get information about the legal procedures
- ***No help***: The [nationality] user shouldn’t help, because the migrant user should find a solution to this problem on his/her own”
- None of the above.

Scenario 7

Problem presented by a migrant user: "I need to analyze my rental agreement, but I do not understand the legal standards in the new country”

As [nationality], please select the solution you think should be recommended to future users of the website:

- ***Dependency:*** The [nationality] user should find legal assistance
- ***Autonomy:*** The [nationality] user should inform and support the migrant user to find legal assistance
- ***No help***: The [nationality] user shouldn’t help, because the migrant user should find a solution to this problem on his/her own”
- None of the above.

Scenario 8

Problem presented by a migrant user: "I need to write a document in the official language of the new country, which I do not speak”

As [nationality], please select the solution you think should be recommended to future users of the website:

- ***Dependency***: The [nationality] user should find an official translator
- ***Autonomy***: The [nationality] user should inform and support the migrant user on how to find an official translator
- ***No help***: The [nationality] user shouldn’t help, because the migrant user should find a solution to this problem on his/her own”
- None of the above.

Scenario 9

Problem presented by a migrant user: “I want to meet and interact with people in the new country.”

As [nationality], please select the solution you think should be recommended to future users of the website:

- ***Dependency:*** The [nationality] user should find the migrant user a social activity or community event for him/her to attend
- ***Autonomy***: The [nationality] user should inform and support the migrant citizen on how to be updated about the social activities and events in the community
- ***No help***: The [nationality] user shouldn’t help, because the migrant user should find a solution to this problem on his/her own”
- None of the above.

Scenario 10

Problem presented by a migrant user: “I would like to travel across the new country”

As [nationality], please select the solution you think should be recommended to future users of the website:

- ***Dependency***: The [nationality] user should get the travel tickets for the migrant user
- ***Autonomy***: The [nationality] user should inform and support the migrant user on how to get the travel tickets
- ***No help:*** The [nationality] user shouldn’t help, because the migrant user should find a solution to this problem on his/her own”
- None of the above.

1.1.8 Helping orientations

Helping Orientations Inventory by Maki et al. (2017)

Please indicate how much you agree or disagree with the following statements, using the scale.
1 Strongly disagree
2 Disagree
3 Somewhat disagree
4 Neither agree nor disagree
5 Somewhat agree
6 Agree
7 Strongly agree

- Teaching migrants to take care of themselves is good for society because it makes them independent.
- The goal of helping should be to make sure migrants can eventually take care of their own needs.
- Helping migrants now makes them better able to solve their own problems in the future.

- I help migrants so that they can learn to solve their own problems.
- Helping migrants is all about making them better able to fix their own problems.
- I like to help migrants develop the skills and knowledge to help themselves.
- Helping migrants makes them better able to solve their own problems.
- When helping migrants, equipping them with knowledge and skills is the most important thing.
- I help migrants because I like solving other people's problems.
- The goal of helping should be to make sure that migrants have their immediate needs met.
- In general, solving migrants' problems for them is good for society because it helps meet immediate needs.
- I like to try to help people even if the issue might come up again.
- I help migrants because they are unable to help themselves.
- All people deserve help equally regardless of their personality and life circumstances.
- I help migrants because we like taking care of people's problems.
- Helping is all about fixing migrants's problems for them.
- Helping migrants only makes them more needy in the future.
- Helping creates a weaker society because migrants will come to depend on others in times of hardship.
- In general, solving migrants' problems for them is bad for society because they come to expect it in the future.
- Teaching migrants to take care of themselves is bad for society because it makes them dependent.
- Helping others now will only make them dependent on others to solve their problems in the future.
- Helping migrants can weaken society because it divides society into those who can help and those who need help.
- Helping migrants makes them less able to solve their own problems.
- Solving migrants 'problems for them makes their situation worse in the long run.
- *This is a control question to screen out random clicking. Please select "disagree" to demonstrate you have read this.*

1.1.9 Sociodemographic information

Instructions
Please enter your mTurk worker ID. Pasting the ID is advised. At the end of the survey, you will be given a unique completion code to insert in MTurk platform. Be sure to enter your completion code correctly to ensure prompt payment.

Age
How old are you? (Please use numbers to represent years)

Sex
- Male
- Female
- I prefer not to answer this question

Migration experience
If you ever lived or you are currently living outside your country, for how long did that experience lasted/lasts?
- I´ve never lived outside my country
- Less than a month
- 1-6 months
- 6-12 months
- 1-3 years
- 3-5 years
- More than 5 years

Educational level
What is the highest level of education you have completed?
- Elementary school
- Junior high school
- High school
- College Associate’s degree
- College Bachelor’s degree
- Graduate/Professional degree
- Don’t know

Political orientation
In politics people sometimes talk of “left” and “right”. Where would you place yourself on this scale?
1 Left
7 Right
Don't know

Employment status
What is your current employment status?
- Student
- Unemployed
- Employed (If chosen, indicate what your profession is) ______________________

- Retired
- Other ____

Migration background
Where did your mother born? _______________________________
Where did your father born? _______________________________

Additional questions

How frequently do you:
1 Never
2 Rarely
3 Sometimes
4 Often
5 Very often

- Travel to foreign countries for short periods (e.g., vacations)
- Interact with migrant citizens
- Hear or use the expression "citizen of the world"
- Hear or use the expression "human"

Perceived financial situation

Which of the descriptions comes closest to how you feel about your household's present income?
- I find it very difficult to live on the present income
- I find it difficult to live on present income
- I am managing with the present income
- I´m living comfortably on the present income
- Don't know

1.1.10 Debriefing

THE END! PLEASE READ THE INFORMATION BELOW
The present study is part of a Ph.D. project "From inclusive identities to inclusive societies: Global human identification and autonomy-oriented prosocial behavior regarding immigrants", funded by Fundação para a Ciência e Tecnologia – Portugal (FCT) and being conducted at Instituto Universitário de Lisboa (ISCTE- IUL).

At the beginning, you were told that this study aims to understand what people think about migration experiences and you were informed about an international website developed on this topic. However, the major goal of this project is to identify the psychosocial processes (e.g., social identification, individual characteristics) that are associated with different forms of helping and prosocial behavior regarding immigrants. This is a common approach in this type of research to avoid biased responses, if the real purpose of the study was known.

Your participation was very important and will help us to better understand the psychosocial processes associated with helping behaviors.

You can request additional information about this study by contacting the research team: Margarida Carmona e Lima, mgfcl@iscte-iul.pt
Thank you for your participation in our study!

Please, click ">>" to get you unique completion code
Here is your unique completion code: ${e://Field/mTurkcode}
Copy this value to paste into MTurk. Be sure to enter your completion code correctly to ensure prompt payment. When you have copied this CODE, please, click ">>" to submit your responses. If you want to let us know about your thoughts, please leave your comments below.

1.2 Additional analysis

1.2.1 Additional information about participants in Study 1

The 168 participants were from 25 different countries, namely: USA (n = 62); Brazil (n = 27); UK (n = 19); India (n = 14); Canada (n = 9); Italy (n = 9); France (n = 4); Germany (n = 3); Portugal (n=2); Romania (n = 2); Spain (n = 2); Trinidad and Tobago (n = 2); Albania (n = 1); Australia (n = 1); Botswana (n = 1); Bulgaria (n = 1); Chile (n = 1); Colombia (n = 1); Egypt (n = 1); Ireland (n = 1); Mexico (n = 1); Pakistan (n = 1); Poland (n = 1); Turkey (n = 1) and Venezuela (n = 1). It is worth noting that migrants represented a minority group in terms of percentage of the total population in all countries: 32.7% of participants were living in countries where international migrants represented less than 5% of the total population (UN, 2019); 22% where international migrants represent 5-15%; and 45.2% where migrants represented more than 15% of the total population. Most participants perceived their financial situation as manageable (56.9%) and comfortable (18.6%), and 24.5% reported difficulties living on their present income.

1.2.2 Group identification: Additional analysis of the measure used in Study 1

Multicomponent Ingroup Identification Scales (Leach et al., 2008) – Reliability

		**Global citizenship identification**	**Human identification**	**National identification**
Unidimensional scale	*M*	4.84	5.22	4.99
	α	.94	.90	.95
SELF-INVESTMENT	*M*	4.92	5.24	5.04
	α	**.94**	**.90**	**.94**
1. Satisfaction	*M*	5.13	5.31	5.06
	α	.87	.85	.91
2. Centrality	*M*	4.60	5.15	4.92
	α	.87	.69	.82
3. Solidarity	*M*	4.97	5.24	5.13
	α	.88	.85	.89
SELF-DEFINITION	*M*	4.63	5.16	4.88
	α	**.86**	**.83**	**.85**
4. Self-Stereotyping	*M*	4.66	5.18	4.82
	r	.67**	.71**	.83**
5. Ingroup Homogeneity	*M*	4.60	5.14	4.93
	r	.70**	.53**	.65**

** $p < .001$

Multicomponent Ingroup Identification Scales (Leach et al., 2008) - EFA

An EFA with Principal Axis Factoring with *oblimin* rotation and Kaiser normalization was conducted to examine whether global citizenship and human identifications are empirically distinct from each other, and from national identification, when measured in a sequential randomized order. Results reveal a three-factor structure, explaining 72.22% of variance (Table 14). All subdimensions of self-investment (i.e., satisfaction, centrality, solidarity) of global citizenship and human identifications loaded together in a single factor (Factor 1), as well as all subdimensions of self-definition (i.e., ingroup homogeneity, self-stereotyping; Factor 3). National identification reproduced the theoretical structure, with all its self-investment and self-definition subdimensions loading together in a unique factor (Factor 2), correlating negatively with the other factors. Results indicate a clear empirical distinction between all-inclusive forms of identification and national identification. However, an empirical distinction between the two forms of all-inclusive identification it is not clear when participants are asked to rate their identification with *citizens of the world* and *humans* sequentially. A clearer distinction can be made at the level of their self-definition and self-investment dimensions.

	Factor		
	**1**	**2**	**3**
Citizen of the world identification: Satisfaction (Self-investment dimension)	.87		
Citizen of the world identification: Solidarity (Self-investment dimension)	.87		
Citizen of the world identification: Centrality (Self-investment dimension)	.82		
Human identification: Solidarity (Self-investment dimension)	.58		
Human identification: Centrality (Self-investment dimension)	.55		
Human identification: Satisfaction (Self-investment dimension)	**.46**	**-.40**	
National identification: Solidarity (Self-investment dimension)		-.91	
National identification: Satisfaction (Self-investment dimension)		-.85	
National identification: Self-stereotyping (Self-definition dimension)		**-.79**	**.33**
National identification: Centrality (Self-investment dimension)		-.74	
National identification: Ingroup homogeneity (Self-definition dimension)		-.59	
Human identification: Ingroup homogeneity (Self-definition dimension)			.79
Human identification: Self-stereotyping (Self-definition dimension)			.71
Citizen of the world identification: Ingroup homogeneity (Self-definition dimension)	**.47**		**.50**
Citizen of the world identification: Self-stereotyping (Self-definition dimension)	**.44**		**.47**

Extraction Method: Principal Axis Factoring. Rotation Method: Oblimin with Kaiser Normalization.

1.2.3 Altruistic orientation: Additional analysis of the measure used in Study 1

Social Value Orientation (SVO) Slider Measure (Murphy et al., 2011) - Descriptive analysis

	M	***SD***	**Min**	**Max**	***n***	**%**
SVO angle	26.88	13.58	-16.26	53.49	168	
**Types**						
Altruist					0	0
Prosocial					110	65.5
Individualist					57	33.9
Competitive					1	0.6

1.2.4 Perceived diversity of superordinate categories: Secondary analysis of Study 1

Perceived diversity of superordinate categories - Scales items

**Perceived diversity of the superordinate category 'citizen of the world'**
1. There is not "the one" typical citizen of the world, but rather many different kinds of citizens of the world
2. One of citizen of the world's characteristics is its great diversity
3. Citizens of the world share a lot of common attributes (Reverse coded)
4. Citizens of the world similarities outweigh the differences (Reverse coded)
**Perceived diversity of the superordinate category 'human'**
1. There is not "the one" typical human, but rather many different kinds of humans
2. One of human's characteristics is its great diversity
3. Humans share a lot of common attributes (Reverse coded)
4. Humans similarities outweigh the differences (Reverse coded)

Perceived diversity of superordinate categories - Descriptive and reliability analysis

	M	*SD*	*α*	**Item 2**	**Item 3**	**Item 4**
**Citizen of the world (4-item scale)**	4.33		.08			
Item 1	5.54	1.31		.18*	-.05	.07
Item 2	5.69	1.22		-	-.38**	-.34**
Item 3 (reverse coded)	2.96	1.23			-	.61**
Item 4 (reverse coded)	3.13	1.39				-
Diversity of Citizens of the World (Items 1 and 2)	5.61	0.97				
Similarity of Citizens of the World (Items 3 and 4 not reverse coded)	4.96	1.18				
**Human (4-item scale)**	4.22		.18			
Item 1	5.50	1.34		.43**	-.12	.02
Item 2	5.79	1.07		-	-.52**	-.15
Item 3 (reverse coded)	2.53	1.20			-	.54**
Item 4 (reverse coded)	3.06	1.45				-
Diversity of Humans (Items 1 and 2)	5.64	1.02				
Similarity of Humans (Items 3 and 4 not reverse coded)	5.21	1.17				

1.2.5 RIP: Additional analysis of the measure used in Study 1

Relative ingroup prototypicality - Descriptive analysis

	M	*SD*	N
**Citizens of the world**			
Mean typicality ratings of characteristic ingroup attributes	4.35	1.38	168
Mean typicality ratings of characteristic outgroup attributes	4.98	1.26	167
Relative ingroup prototypicality for 'citizen of the world'	-0.62	1.48	167
**Humans**			
Mean typicality ratings of characteristic ingroup attributes	4.83	1.48	168
Mean typicality ratings of characteristic outgroup attributes	4.93	1.46	167
Relative ingroup prototypicality for 'human'	-0.09	1.37	167

1.2.6 Helping preferences: Additional analysis of the measure used in Study 1

Helping preferences: Frequencies and MCA

We expected that the options a) and b) would be perceived as dependency-oriented responses and autonomy-oriented responses, respectively. To verify whether the options indeed matched with different patterns of helping responses independently of the scenario's content, we performed a multiple correspondence analysis (MCA). The options "No help" and "None of the above" were defined as missing values to run the MCA. The MCA revealed one relevant dimension accounting for 23.24% of the total variance, with acceptable reliability (α = .73; Kline, 2011), confirmed by discrimination measures, and revealed privileged associations within the dependency-oriented responses, as well as autonomy-oriented responses.

**Discrimination measures**	***N***	**Dim. 1**
Scenario nº 1 - **"I need to make an appointment in a health facility"**		.422
**Dependency:** "[...] should contact the health facility and make the appointment for the migrant user"	18	
**Autonomy:** "[...] should inform and support the migrant user on how to identify a health facility and how to make an appointment"	133	
**No help:** "[...] shouldn't help, because the migrant user should find a solution to this problem on his/her own"	15	
None of the above solution should be recommended to the website users.	2	
Scenario nº 2 -**"I have an appointment with the school teacher of my children, but we don't speak a common language."**		.219
**Dependency:** "[...] should find an official interpreter to be present in the meeting."	24	
**Autonomy:** "[...] should inform and support the migrant user on how to find an official interpreter to be present at the meeting	121	
**No help**: "[...] shouldn't help […]"	19	
None of the above solution should be recommended to the website users.	4	
Scenario nº 3 - **"I would like to bring my family to the new country I'm living in".**		.323
**Dependency:** "[...] should contact the appropriate government services to get information about the legal procedures"	20	
**Autonomy:** "[...] should inform and support the migrant user regarding the appropriate government services to get information about the legal procedures"	133	
**No help:** "[...] shouldn't help […]"	13	
None of the above solution should be recommended to the website users.	2	
Scenario nº 4 - **"I was a victim of discrimination."**		.094
**Dependency**: "[...] should report the incident to the legal authorities"	37	
**Autonomy:** "[...] should inform and support the migrant user on how to report the incident to the legal authorities"	114	
**No help:** "[...] shouldn't help […]"	15	
None of the above solution should be recommended to the website users.	2	
Scenario nº 5 - **"I need to create a CV to apply to job in the new country."**		.429
**Dependency:** "[...] should prepare the CV for the migrant user"	22	
**Autonomy:** "[...] should inform and support the migrant user on how to prepare a good CV"	127	
**No help:** "[...] shouldn't help […]"	17	
None of the above solution should be recommended to the website users.	2	
Scenario nº 6 - **"I have a tourist visa, but I want to obtain a residence permit."**		.395
**Dependency:** "[...] should contact the appropriate government services to get information about the legal procedures"	28	
**Autonomy:** "[...] should inform and support the migrant user regarding the appropriate government services to get information about the legal procedures"	123	
**No help:** "[...] shouldn't help […]"	13	
None of the above solution should be recommended to the website users.	4	

Scenario nº 7 - **“I need to analyze my rental agreement, but I do not understand the legal standards in the new country."**		.269
**Dependency:** “[...] should find legal assistance”	20	
**Autonomy:** “[...] should inform and support the migrant user to find legal assistance”	133	
**No help:** “[...] shouldn’t help […]”	12	
None of the above solution should be recommended to the website users.	3	
Scenario nº 8 - **“I need to write a document in the official language of the new country, which I do not speak.”**		.134
**Dependency:** “[...] should find an official translator”	22	
**Autonomy:** “[...] should inform and support the migrant user on how to find an official translator”	124	
**No help:** “[...] shouldn’t help […]”	16	
None of the above solution should be recommended to the website users.	6	
Scenario nº 9 - **“I want to meet and interact with people in the new country.”**		.244
**Dependency:** “[...] should find the migrant user a social activity or community event for him/her to attend”	42	
**Autonomy:** “[...] should inform and support the migrant citizen on how to be updated about the social activities and events in the community”	113	
**No help:** “[...] shouldn’t help […]”	12	
None of the above solution should be recommended to the website users.	1	
Scenario nº 10 - **“I would like to travel across the new country.”**		.381
**Dependency:** “[...] should get the travel tickets for the migrant user”	11	
**Autonomy:** “[...] should inform and support the migrant user on how to get the travel tickets”	132	
**No help:** “[...] shouldn’t help […]”	20	
None of the above solution should be recommended to the website users.	5	

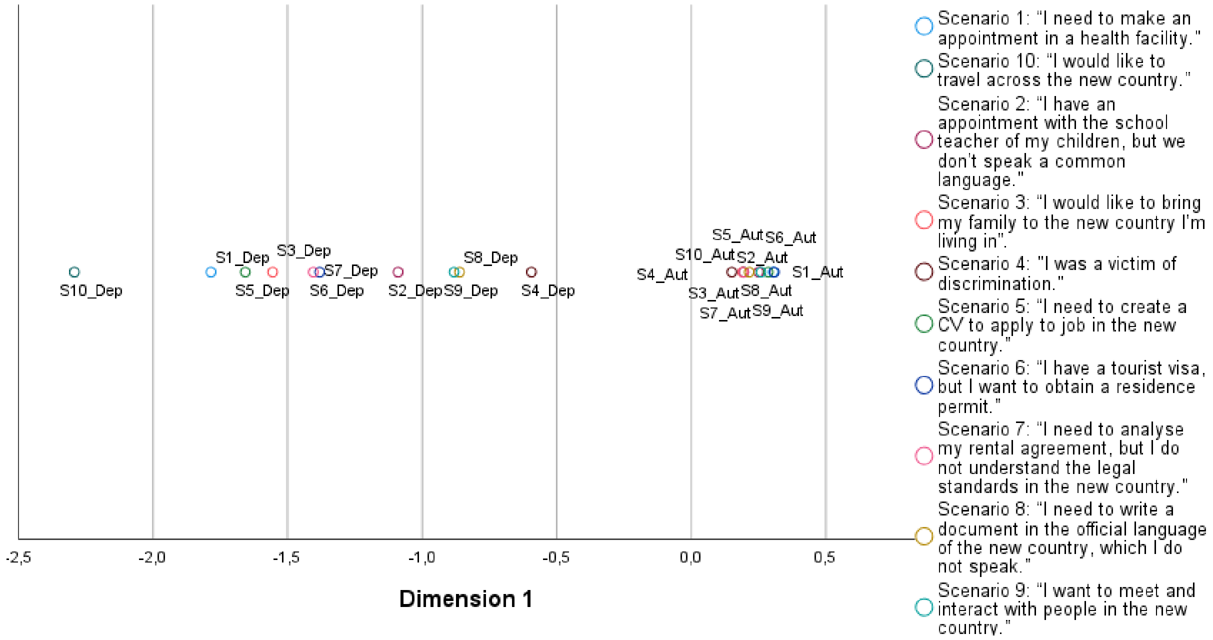

Supplementary Figure 1. Helping preferences Scale: Topological configuration of helping preferences - Dependency-oriented and autonomy-oriented responses

1.2.7 Helping orientations: Additional analysis of the measure used in Study 1

We conducted an EFA with Principal Axis Factoring with *oblimin rotation* and Kaiser normalization to examine the theoretical dimensions on our sample. Scree plot analysis determined the number of retained factors, and pattern matrices were examined for factor loadings (Costello & Osborne, 2005). An initial three-factor solution, explaining 58.87% of the variance was obtained, reproducing the proposed theoretical dimensions. However, three items showed cross-loadings (i.e., 12, 14, and 16), in Step 1. After removing these items, a final three-factor solution was obtained, in Step 2, explaining 60.76% of the variance, with acceptable reliability.

Exploratory factor pattern matrix – Step 1

	1	*2*	*3*
1 (2/aut): Teaching migrants to take care of themselves is good for society because it makes them independent.	.74		
2 (5/aut): The goal of helping should be to make sure migrants can eventually take care of their own needs.	.72		
8 (30/aut): When helping migrants, equipping them with knowledge and skills is the most important thing.	.71		
4 (16/aut): I help migrants so that they can learn to solve their own problems.	.68		
5 (17/aut): Helping migrants is all about making them better able to fix their own problems.	.64		
6 (24/aut): I like to help migrants develop the skills and knowledge to help themselves.	.63		
3 (10/aut): Helping migrants now makes them better able to solve their own problems in the future.	.60		
7 (29/aut): Helping migrants makes them better able to solve their own problems.	.59		
14 (23/dep): All people deserve help equally regardless of their personality and life circumstances.	**.42**	**.31**	
11 (19/dep): In general, solving migrants' problems for them is good for society because it helps meet immediate needs.		.66	
9 (8/dep): I help migrants because I like solving other people's problems.		.66	
15 (25/dep): I help migrants because we like taking care of people's problems.		.58	
10 (11/dep): The goal of helping should be to make sure that migrants have their immediate needs met.		.56	
13 (22/dep): I help migrants because they are unable to help themselves.		.47	
12 (21/dep): I like to try to help people even if the issue might come up again.	**.41**	**.45**	
16 (32/dep): Helping is all about fixing migrants's problems for them.		**.43**	**.40**
17 (4/opp): Helping migrants only makes them more needy in the future.			.85
19 (7/opp): In general, solving migrants' problems for them is bad for society because they come to expect it in the future.			.84
23 (26/opp): Helping migrants makes them less able to solve their own problems.			.84
24 (27/opp): Solving migrants 'problems for them makes their situation worse in the long run.			.78
22 (20/opp): Helping migrants can weaken society because it divides society into those who can help and those who need help.			.77
18 (6/opp): Helping creates a weaker society because migrants will come to depend on others in times of hardship.			.75
21 (13/opp): Helping others now will only make them dependent on others to solve their problems in the future.			.72
20 (9/opp): Teaching migrants to take care of themselves is bad for society because it makes them dependent.			.66

Exploratory factor pattern matrix - Step 2

	1	*2*	*3*	*α*	*M*
**Orientation to opposition to helping**				.93	3.09
17 (4/opp): Helping migrants only makes them more needy in the future.	.85				
19 (7/opp): In general, solving migrants' problems for them is bad for society because they come to expect it in the future.	.84				
23 (26/opp): Helping migrants makes them less able to solve their own problems.	.83				
24 (27/opp): Solving migrants 'problems for them makes their situation worse in the long run.	.78				
22 (20/opp): Helping migrants can weaken society because it divides society into those who can help and those who need help.	.77				
18 (6/opp): Helping creates a weaker society because migrants will come to depend on others in times of hardship.	.74				
21 (13/opp): Helping others now will only make them dependent on others to solve their problems in the future.	.71				
20 (9/opp): Teaching migrants to take care of themselves is bad for society because it makes them dependent.	.65				
**Orientation for dependency**				.76	4.38
11 (19/dep): In general, solving migrants' problems for them is good for society because it helps meet immediate needs.		.68			
9 (8/dep): I help migrants because I like solving other people's problems.		.66			
10 (11/dep): The goal of helping should be to make sure that migrants have their immediate needs met.		.56			
15 (25/dep): I help migrants because we like taking care of people's problems.		.56			
13 (22/dep): I help migrants because they are unable to help themselves.		.48			
**Orientation for autonomy**				.88	5.51
1 (2/aut): Teaching migrants to take care of themselves is good for society because it makes them independent.			.73		
2 (5/aut): The goal of helping should be to make sure migrants can eventually take care of their own needs.			.72		
8 (30/aut): When helping migrants, equipping them with knowledge and skills is the most important thing.			.70		
4 (16/aut): I help migrants so that they can learn to solve their own problems.			.66		
5 (17/aut): Helping migrants is all about making them better able to fix their own problems.			.64		
6 (24/aut): I like to help migrants develop the skills and knowledge to help themselves.			.62		
3 (10/aut): Helping migrants now makes them better able to solve their own problems in the future.			.61		
7 (29/aut): Helping migrants makes them better able to solve their own problems.			.58		

1.2.8 Zero-order correlations among all variables of Study 1

	1	2	3	4	5	6	7	8	9	10	11	12	13	14	15	16	17	18	19	20	21
1. Global ident.: Self-investment	-																				
2. Human ident: Self-investment	.71**																				
3. Global ident.: Self-definition	.65**	.56**																			
4. Human ident: Self-definition	.39**	.46**	.62**																		
5. Pref. for helping in general	.21**	.26**	.17*	.25**																	
6. Orient. to opposition to helping	.01	.02	.07	-.06	-.44**																
7. Orientation for dependency	.37**	.32**	.35**	.34**	.29**	.13															
8. Preference for autonomy rel. dep	-.09	-.03	-.07	-.07	.18*	-.39**	-.35**														
9. Orientation for autonomy	.32**	.34**	.22**	.29**	.62**	-.28**	.40**	.17*													
10. National identification	.44**	.62**	.37**	.31**	.04	.25**	.26**	-.09	.16*												
11. Altruistic orientation	.07	.03	-.01	-.05	.20**	-.25**	-.03	.09	.19*	-.13											
12. RIP for citizens of the world	.04	.10	.11	-.01	-.07	.24**	-.04	.02	-.14	.21**	-.18*										
13. RIP for humans	.13	.12	.18*	.13	.10	.03	.10	-.09	.10	.11	-.10	.42**									
14. Age	-.02	-.02	-.05	-.10	-.09	-.16*	-.11	.14	-.08	-.07	-.12	-.03	-.07								
15. Migration experience	.24**	.11	.08	-.02	-.05	.19*	.08	-.21**	.06	.17*	.02	.00	-.02	.11							
16. Educational level	.11	.05	.10	.01	.02	.14	.08	-.14	.03	.12	-.20*	.03	.04	.07	.28**						
17. Political orientation	-.09	-.03	-.09	-.08	-.43**	.59**	.04	-.35**	-.36**	.22**	-.20*	.16*	.07	-.01	.11	.13					
18. Freq. of traveling	.04	.02	-.05	-.08	-.04	.24**	.07	-.12	.01	.20*	-.04	.13	-.05	.03	.35**	.34**	.13				
19. Freq. of interaction with migrants	.19*	.16*	.16*	.13	.10	.10	.23**	-.19*	.11	.17*	.23**	-.02	.08	-.14	.28**	.17*	.04	.34**			
20. Freq. of hearing or using the expression "citizen of the world"	.38**	.22**	.29**	.19*	-.02	.34**	.24**	-.31**	.07	.16*	-.01	.06	.03	.04	.25**	.11	.06	.26**	.35**		
21. Freq. of hearing or using the expression "human"	.37**	.38**	.23**	.20*	.08	.06	.09	-.10	.15*	.20*	.01	-.06	-.06	.15*	.20**	.07	-.10	.08	.18*	.49**	
22. Perceived financial situation	.08	.06	.09	-.07	-.06	.04	-.06	.04	-.05	.03	-.04	.17*	.15	-.06	.04	.16*	.00	.08	.02	-.08	.03

1.2.9 RIP: Examining the preconditions for ingroup projection in Study 1

Regarding relative ingroup prototypicality, we first examined the preconditions for ingroup projection to occur by analyzing if the mean scores for national identification, global citizenship, and human identification were above the scale midpoint. For ingroup projection to occur, participants should identify both with their ingroup (i.e., national group) and the superordinate groups (i.e., citizens of the world; humans). One sample t-tests showed means significantly above the scale midpoint of 4 for national identification, M = 4.99, SD = 1.20, t(167) = 10.667, p < .001; global citizenship self-investment M = 4.92, SD = 1.15, t(167) = 10.415, p < .001, and self-definition dimensions, M = 4.63, SD = 1.26, t(167) = 6.497, p < .001; and for human identification self-investment M = 5.24, SD = 1.07, t(167) = 15.068, p < .001, and self-definition dimensions, M = 5.16, SD = 1.17, t(167) = 12.878, p < .001. These results indicate that preconditions for the occurrence of ingroup projection were satisfied. It is worth noting that participants identified themselves more strongly as humans than as citizens of the world both at self-investment, t(167) = 4.854, p < .001, and self-definitions dimensions, t(167) = 6.474, p < .001.

1.2.10 Predicting helping preferences by type in Study 1

We conducted two multiple regressions for *preference for autonomy- relative to dependency-oriented help* (models 5 and 6).

	**Preference for autonomy- relative to dependency-oriented help**						
		95% CI for *B*					
	B	*LL*	*UL*	*SE B*	β	R^2	ΔR^2
**Model 5 (Self-investment)**						.15	.13
Constant	1.03***	0.86	1.20	0.09			
Political orientation	-0.04***	-0.06	-0.02	0.01	-.37***		
Global citizenship ident.: SI	-0.04$^+$	-0.07	0.00	0.02	-.21$^+$		
Human identification: SI	0.03	-0.01	0.07	0.02	.14		
**Model 6 (Self-definition)**						.14	.12
Constant	1.04***	0.88	1.21	0.08			
Political orientation	-0.04***	-0.06	-0.02	0.01	-.36***		
Global citizenship ident.: SD	-0.02	-0.05	0.01	0.02	-.12		
Human identification: SD	0.01	-0.03	0.04	0.02	.04		

Regarding *preference for autonomy- relative to dependency-oriented help*, the model for self-investment (model 5) was statistically significant (R^2 = .146, F(3, 148) = 8.443, p < .001; adjusted R^2 = .129). However, only political orientation was negatively related to *preference for autonomy- relative to dependency-oriented help;* self-investment as *citizens of the world* only approached significance (p = .059). Similarly, the model for self-definition (model 6) was statistically significant (R^2 = .135, F(3, 148) = 7.730, p < .001; adjusted R^2 = .118), however, only political orientation negatively predicted *preference for autonomy- relative to dependency-oriented help*. No significant effects were found for self-definition as a *citizen of the world* or *human*.

2 Study 2

2.1 Full protocol

Study 2: List and order of measures
Informed consent
Eligibility: Nationality and country of residence
Experimental manipulation and manipulation check
Group identification
Helping preferences
Willingness to help
Costs and benefits of helping
Helping orientations
Willingness to participate in collective action
Feelings towards migrants
Relative prototypicality
Entitativity
Essentialism
Group representations
Migrants' origin
Social Dominance Orientation
National identification
Sociodemographic information
Debriefing / Completion code

2.1.1 Informed consent

Welcome to our study!
ISCTE-University Institute of Lisbon (Portugal) is studying how people use online platforms. You will watch videos, and your opinion about related topics will be asked. In total, this should take approximately 20 minutes.
For the specific purpose of this study, there are 2 conditions to be eligible to participate, and be paid:
1) You must be fluent in English
2) You must be currently living in the country in which you and your parents were born
If you do not meet these conditions, please do not fill out the survey.
We ask you to fill out all the questions. We are interested in the first answer that comes to your mind, there are no right or wrong answers. The information that you provide will not be used to judge you in any way, and this research follows the recommendations of the ISCTE-IUL Ethics Committee.
If you want more information, now or in the future, you are free to contact the researchers by e-mail (mgfcl@iscte-iul.pt). Thank you! The research team,

Please read the following consent: I am aged 18 years or older. I agree to voluntarily participate in this study. I am free to withdraw at any time, without giving a reason. If my results are used in scientific publications, or are published in any other way, my data will be completely anonymous. My data will

not be sent to third parties. Only researchers will have access to data. There are no physical, legal or economic risks associated with participating in this study.

To ensure your payment: 1) Along the survey you will find control questions to screen out random clicking. You must carefully read all the questions and respond correctly to the control questions. If you fail to respond correctly to control questions, you will not receive your completion code/payment; 2) At the end of the survey you will be given a completion code to copy and paste into Clickworker platform. Be sure to enter your completion code correctly to ensure payment.

I confirm that I have read and understood the above and freely consent to participating in this study. Multiple choice question: Yes/No (If "no" is select, participant will be automatically directed to the end of the survey)
Yes / No

I confirm that I'm fluent in English.
Yes / No

I confirm that I am currently living at the country where me and my parents were born.
Yes / No

2.1.2 Eligibility: Nationality and country of residence

CAPTCHA Test (Robot check)

Nationality (A to Z). (If you hold multiple nationalities, please select the one with which you identify the most. This is a long A- Z menu. If your nationality is missing in the list, please select "Other" at the bottom and then type it in the text box that will appear next)
List of countries similar to Study1

Country where you and your parents were born, and where you are currently living in (This is a long A- Z menu. If your country is missing in the list, please select "Other" at the bottom and then type it in the text box that will appear next)
List of countries similar to Study1

2.1.3 Experimental manipulation

Instructions
This section of the study analyses a platform of online learning. Please watch carefully this 2-minute video. Please, click on EXPANSION SCREEN button and then press PLAY button to start.

Video content
In this study, our goal is to better understand online learning. We aim to understand if a voice over in online presentations helps to retain information more effectively. Please imagine that you are taking an online Psychology course. You will watch a short presentation explaining an important concept in this scientific field. Some participants will see a presentation using a voice over, that is, they will hear a narrator - a person who provides a voice over - and others will see a presentation without it. You will be asked a few questions at the end. Please pay full attention to the information you will see, without

interruptions. You don’t need to have any prior knowledge in Psychology, nor will you receive a grade. However, we will ask you a few brief questions about the content and what you thought of the material at the end.
Please note that you have been randomly assigned to a presentation without a voice over. That is, you will not hear a narrator.

The presentation will start now:
“One important concept in Psychology is identification with groups. This happens when people see themselves as members of a group. Groups can be small and involve only a few people, or larger and involve many more people, and ultimately everyone. An example of a large group with whom people can identify with is **citizens of the world (condition 1)/ humans (condition 2)/ daughters and sons (control)**. When you identify with **citizens of the world/humans/daughters and sons**, you think that you have a lot in common and you are similar to other **citizens of the world/humans/daughters and sons**; you feel that being a **citizen of the world/human/daughters or a son** is an important part of who you are, your identity; you have a good feeling about it; and you feel solidarity with other **citizens of the world/humans/daughters and sons**.”
The presentation is over.

Video’ shots (example for citizens of the world condition):

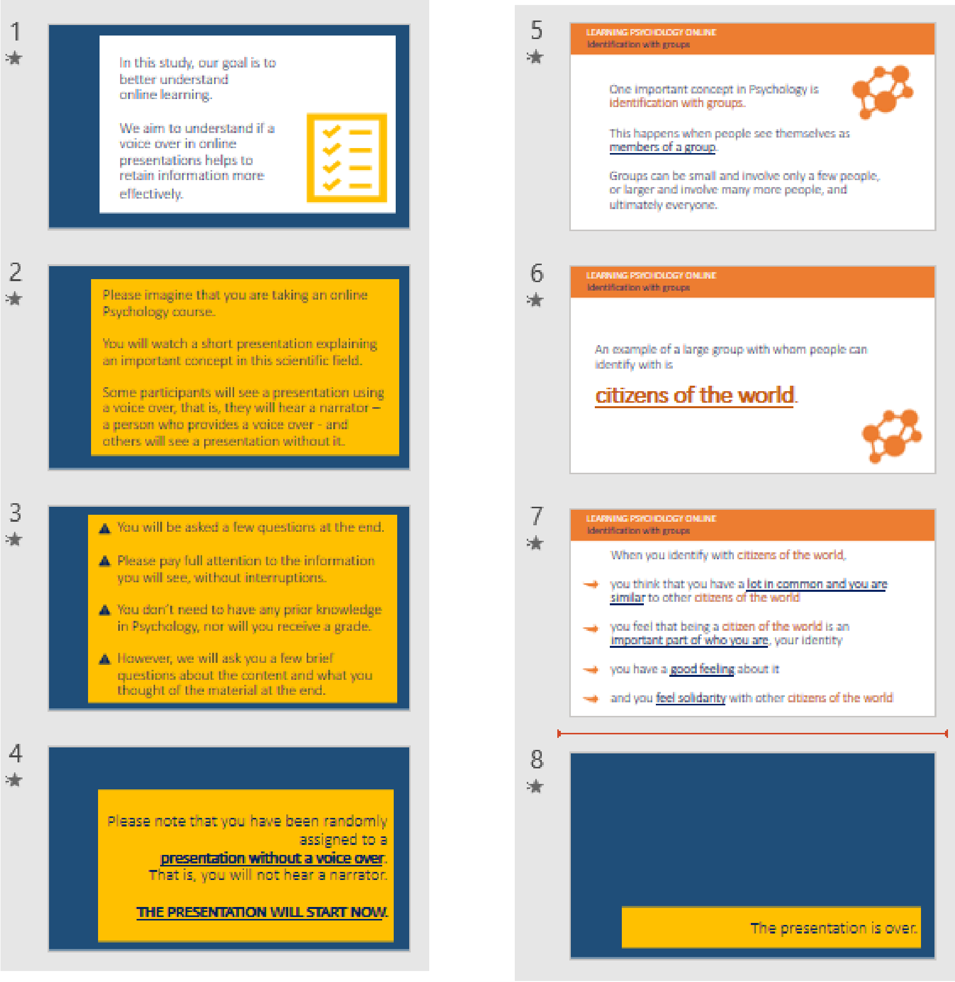

2.1.4 Manipulation check

Thank you for watching. Please answer a few questions about the presentation.

Please choose the appropriate word, from the list below, to complete the following sentence: “The concept referred in the presentation describes what happens when people see themselves as members of a _________”:

- Company
- Group
- Sports team

Which example was given to exemplify the concept, in the presentation?
Open question

Please characterize the voice over you have heard during the presentation.

- I heard a female voice over
- I heard a male voice over
- I did not hear any voice over

Please describe, in a few words, what does it mean to you to belong to the group of citizens of the world (condition 1)/ humans (condition 2)/ daughters and sons (control), and how important (or not) it is to you.
Open question

2.1.5 Group identification

Please indicate how much you agree or disagree with the following statements, using the scale.
1 Strongly disagree
2 Disagree
3 Somewhat disagree
4 Neither agree nor disagree
5 Somewhat agree
6 Agree
7 Strongly agree

Single item from Postmes et al. (2013)

- I identify with ${e://Field/Condition}.

Multicomponent Ingroup Identification Scale by Leach et al. (2008)

- I have a lot in common with the average ${e://Field/Condition}.
- I am similar to the average ${e://Field/Condition}.
- I feel a bond with ${e://Field/Condition}.
- I feel solidarity with ${e://Field/Condition}.
- I feel committed to ${e://Field/Condition}.
- *This is a control question to screen out random clicking. Please select "disagree" to demonstrate you have read this.*

2.1.6 Helping preferences

Instructions
This section of the study analyses a platform of online interaction. Please watch carefully this 1-minute video. Please, click on EXPANSION SCREEN button and then press PLAY button to start.

Video content

"Recently, a new international website was launched, where migrants can chat with nationals to find solutions to problems they encounter. Website users can sign up as MIGRANTS (requesting help) and NATIONALS (providing help). Please imagine you have signed up as a national user and received requests from migrant users to provide help. In the next pages, you will find different requests and a list of possible solutions for each request, which were previously suggested by other users. Please select the solution you think you would most likely adopt, and that you would recommend to future users of the website. Please keep in mind that all the solutions can be easily implemented in an online interaction between people."

Scenario 1
Problem presented by a migrant user: "**I need to make an appointment in a health facility**."
Please select the solution you think you would most likely adopt if you were a [nationality] user, and that you would recommend to future users of the website

- ***Dependency*:** I would contact the health facility and make the appointment, for the migrant user.
- ***Autonomy*:** I would explain to the migrant user how to identify a health facility and how to make an appointment.
- ***No help*:** I would/could not help the migrant user in relation to this issue.

Scenario 2
Problem presented by a migrant user: "**I have an appointment with the school teacher of my children, but we don't speak a common language**."
Please select the solution you think you would most likely adopt if you were a [nationality] user, and that you would recommend to future users of the website

- ***Dependency:*** I would find an official interpreter to be present at the meeting, for the migrant user.
- ***Autonomy*:** I would explain to the migrant user how to find an official interpreter to be present at the meeting.
- ***No help:*** I would/could not help the migrant user in relation to this issue.

Scenario 3
Problem presented by a migrant user: "**I would like to bring my family to the new country I'm living in**".
Please select the solution you think you would most likely adopt if you were a [nationality] user, and that you would recommend to future users of the website

- ***Dependency*:** I would contact the appropriate government services to get information about the legal procedures, for the migrant user.
- ***Autonomy*:** I would explain to the migrant user how to contact the appropriate government services to get information about the legal procedures.

- ***No help:*** I would/could not help the migrant user in relation to this issue.

Scenario 4
Problem presented by a migrant user: "**I was a victim of discrimination**."
Please select the solution you think you would most likely adopt if you were a [nationality] user, and that you would recommend to future users of the website

- ***Dependency*****:** I would report the incident to the legal authorities, for the migrant user.
- ***Autonomy:*** I would explain to the migrant user how to report the incident to the legal authorities.
- ***No help*****:** I would/could not help the migrant user in relation to this issue.

Scenario 5
Problem presented by a migrant user: "**I need to create a CV to apply to a job in the new country**."
Please select the solution you think you would most likely adopt if you were a [nationality] user, and that you would recommend to future users of the website

- ***Dependency*****:** I would prepare a good CV model for the migrant user.
- ***Autonomy*****:** I would explain to the migrant user how to prepare a good CV.
- ***No help*****:** I would/could not help the migrant user in relation to this issue.

Scenario 6
Problem presented by a migrant user: "I have a tourist visa, but I want to obtain a residence permit."
Multiple choice question: Please select the solution you think you would most likely adopt if you were a [nationality] user, and that you would recommend to future users of the website

- ***Dependency*****:** I would contact the appropriate government services to get information about the legal procedures, for the migrant user.
- ***Autonomy*****:** I would explain to the migrant user how to contact the appropriate government services to get information about the legal procedures.
- ***No help*****:** I would/could not help the migrant user in relation to this issue.

Scenario 7
Problem presented by a migrant user: "**I need to analyse my rental agreement, but I do not understand the legal standards in the new country**."
Please select the solution you think you would most likely adopt if you were a [nationality] user, and that you would recommend to future users of the website

- ***Dependency:*** I would find legal assistance for the migrant user.
- ***Autonomy*****:** I would explain to the migrant user how to find legal assistance.
- ***No help*****:** I would/could not help the migrant user in relation to this issue.

Scenario 8
Problem presented by a migrant user: "**I need to write a document in the official language of the new country, which I do not speak**."
Please select the solution you think you would most likely adopt if you were a [nationality] user, and that you would recommend to future users of the website
Dependency: I would find an official translator, for the migrant user.
Autonomy: I would explain to the migrant user how to find an official translator.
No help: I would/could not help the migrant user in relation to this issue.

Scenario 9
Problem presented by a migrant user: "**I want to meet and interact with people in the new country.**"
Please select the solution you think you would most likely adopt if you were a [nationality] user, and that you would recommend to future users of the website
Dependency: I would find a social activity or community event for the migrant user to attend.
Autonomy: I would explain to the migrant user how to be updated about the social activities and events in the community.
No help: I would/could not help the migrant user in relation to this issue.

Scenario 10
Problem presented by a migrant user: "**I want to travel in the new country**."
Please select the solution you think you would most likely adopt if you were a [nationality] user, and that you would recommend to future users of the website
Dependency: I would get the travel tickets for the migrant user.
Autonomy: I would explain to the migrant user how to get the travel tickets.
No help: I would/could not help the migrant user in relation to this issue.

If you want to comment your choices, please use the space below.
Open question

2.1.7 Willingness to help

Please indicate how much you agree or disagree with the following statement, using the scale. Likert scale: 1 (not at all) to 7 (very much). To what extent do you see yourself using this website and helping migrants, in your daily life?
1 = Not at all
7 = Very much

2.1.8 Costs and benefits of helping

Please think about the costs and benefits of helping a migrant who is living in your country of residence. Please rate to what extent helping would represent a cost or a benefit, using the slide below.

Helping a migrant, who is living in [country of residence], would represent…
1= More costs than benefits to myself
7 = More benefits than costs to myself

Helping a migrant, who is living in [country of residence], would represent…
1= More costs than benefits to [country of residence]
7 = More benefits than costs to [country of residence]

Helping a migrant, who is living in [country of residence], would represent…
1= More costs than benefits to the migrant
7 = More benefits than costs to the migrant

2.1.9 Helping orientations

Same as in Study 1
Items dropped out in Study 2 from orientation for dependency scale:

- "I like to try to help people even if the issue might come up again."
- "All people deserve help equally regardless of their personality and life circumstances."
- "Helping is all about fixing migrants' problems for them."

2.1.10 Willingness to participate in collective action

Please indicate how much you agree or disagree with the following statements, using the scale.
1 = Strongly disagree
2 = Disagree
3 = Somewhat disagree
4 = Neither agree nor disagree
5 = Somewhat agree
6 = Agree
7 = Strongly agree

- I would sign a petition that demands civic and political rights for migrants to ensure their integration in society, for example the right to vote or to became members of political parties.
- I would sign a petition that demands social and economic rights for migrants to ensure their integration in society, for example protection against poverty and the right to housing.

2.1.11 Feelings towards migrants

Below is something that looks like a thermometer. We call it a 'feeling thermometer' because it measures your feelings towards others. Here's how it works. If you don't know too much about a group of people, or don't feel particularly warm or cold towards them, then you should place the thermometer in the middle, at the 50-degree mark. If you have a warm feeling, or feel favourably towards them, you would give it a score somewhere between 50 and 100 depending on how warm your feeling is. On the other hand, if you don't feel very favourably, or if you don't care for too much about that people, then you would place the mark somewhere between the 0 and 50-degree mark
How do you feel towards migrants who are living in [country of residence]?
0 = Cold
50
100 = Warm

2.1.12 Relative prototypicality

Please indicate how much you agree or disagree with the following statement, using the scale.
1 = Strongly disagree
2 = Disagree
3 = Somewhat disagree
4 = Neither agree nor disagree
5 = Somewhat agree
6 = Agree
7 = Strongly agree

- [Nationality] are prototypical [citizens of the world/ humans/ daughters and sons].
- Migrants are prototypical [citizens of the world/ humans/ daughters and sons].

2.1.13 Entitativity

Please think about [citizens of the world/ humans/ daughters and sons] as a social category. For each question below, we would like you to rate the category [citizens of the world/ humans/ daughters and sons], using the rating scale provided for each question.

To what extent do ${e://Field/Condition} 'not qualify at all as a group' or 'very much qualify as a group'?
1 = Not qualify at all as a group
7 = Very much qualify as a group

In some categories, people interact very much with one another. In some, there is almost no interaction between members of the category. To what extent do [citizens of the world/ humans/ daughters and sons] 'not interact at all with one another' or 'interact very much with one another'?
1 = Not interact at all with one another
7 = Interact very much with one another

Some categories are very important in the eyes of the people that are part of it. Some have no importance at all in their eyes. To what extent is the category do [citizens of the world/ humans/ daughters and sons] 'not at all important' or 'very much important' to the people that are part of it?
1 = Not at all important
7 = Very much important

In some categories, members of the category share with one another common fate. In some categories, members are not linked by a common fate. To what extent do [citizens of the world/ humans/ daughters and sons] 'not share a common fate' or 'share a common fate'?
1 = Not share a common fate
7 = Share a common fate

In some categories, members of the category pursue common goals. In some categories, members are not linked by any common goals. To what extent do [citizens of the world/ humans/ daughters and sons] 'not have common goals' or 'pursue common goals'?
1 = Not have common goals
7 = Pursue common goals

Some categories allow people to make many judgments about their members; in other words, knowing that someone belongs to the category tells us a lot about that person, meaning that membership is very informative. Other categories only allow a few judgments about their members, meaning that membership is not very informative. To what extent knowing that someone belongs to the category [citizens of the world/ humans/ daughters and sons] 'is not very informative' or 'tells a lot about that person'?
1 = Is not very informative
7 = Tells a lot about that person

Some categories contain members who are very similar to one another; they have many things in common. Other categories contain members who differ greatly from one another, and don't share many characteristics. To what extent are [citizens of the world/ humans/ daughters and sons] 'diverse' or 'similar' to one another?
1 = Diverse
7 = Similar

2.1.14 Essentialism

Some categories have sharper boundaries than others. For some, membership is clear-cut, definite, and of 'either/or' variety; people belong to the category or they do not. For others, membership is more 'fuzzy'; people belong to the category in varying degrees. To what extent belonging to [citizens of the world/ humans/ daughters and sons] is 'clear-cut' or 'fuzzy'?
1 = Clear-cut
7 = Fuzzy

Some categories are more natural than others, whereas others are more artificial. To what extent is the category of [citizens of the world/ humans/ daughters and sons] more 'artificial' or more 'natural'?
1 = Artificial
7 = Natural

Membership in some categories is easy to change; it is easy for group members to become non-members. Membership in other categories is relatively immutable; it is difficult for category members to become non-members. To what extent is belonging to [citizens of the world/ humans/ daughters and sons] 'easily changed' or 'not easily changed'?
1 = Easily changed
7 = Not easily changed

Some categories are more stable over time than others; they have always existed, and their characteristics have not changed much throughout history. Other categories are less stable; their characteristics have changed substantially over time, and they may not always have existed. To what degree do [citizens of the world/ humans/ daughters and sons] 'change much over time' or 'change little over time'?
1 = Change much over time
7 = Change little over time

Some categories have necessary features or characteristics; without these characteristics someone cannot be a category member. Other categories have many similarities, but no features or characteristics are necessary for membership. To what extent do [citizens of the world/ humans/ daughters and sons] 'have necessary characteristics' or 'do not have necessary characteristics' to be members?
1 = Have necessary characteristics
7 = Do not have necessary characteristics

Some categories have an underlying reality, which means that although members have similarities and differences on the surface, underneath they are basically the same. Other categories also have many similarities and differences on the surface, but do not have an underlying sameness. To what extent do

[citizens of the world/ humans/ daughters and sons] 'have an underlying sameness' or 'do not have an underlying sameness'?
1 = Have an underlying sameness
7 = Do not have an underlying sameness

2.1.15 Perceptions of choice

Membership in some categories is the result of an individual choice to belong to a group. Other categories do not have this element of choice; members must be born into the group or possess certain predefined characteristics outside of their control that will determine their membership. To what extent belonging to the group of [citizens of the world/ humans/ daughters and sons] 'is the result of a choice' or 'does not result from a choice'?
1 = Is the result of a choice
7 = Does not result from a choice

2.1.16 Evaluative status and valence

Please indicate how much you agree or disagree with the following statement, using the scale.
1 = Strongly disagree
2 = Disagree
3 = Somewhat disagree
4 = Neither agree nor disagree
5 = Somewhat agree
6 = Agree
7 = Strongly agree

- Generally speaking, people highly respect and admire [citizens of the world/ humans/ daughters and sons].
- Generally speaking, people have a positive image of [citizens of the world/ humans/ daughters and sons].

2.1.17 Perceptions of group size

- The group of [citizens of the world/ humans/ daughters and sons] includes every person on Earth.
- *This is a control question to screen out random clicking. Please select "disagree" to demonstrate you have read this.*

2.1.18 Group representations

Please indicate how much you agree or disagree with the following statement, using the scale.
1 = Strongly disagree
2 = Disagree
3 = Somewhat disagree
4 = Neither agree nor disagree

5 = Somewhat agree
6 = Agree
7 = Strongly agree

- When I think of migrants and [Nationality], who are living in [country of residence], I see them as one group.
- When I think of migrants and [Nationality], who are living in [country of residence], I see them as two separate groups.
- When I think of migrants and [Nationality], who are living in [country of residence], I see them as two groups on the same team.

2.1.19 Migrant's origin

When you think about migrants who are living in [country of residence], where are they from?

- East Asia and Pacific
- Europe and Central Asia
- Latin America and Caribbean
- Middle East and North Africa
- North America
- South Asia
- Sub-Saharan Africa

2.1.20 Social dominance orientation

Please indicate how much you agree or disagree with the following statement, using the scale.
1 = Strongly disagree
2 = Disagree
3 = Somewhat disagree
4 = Neither agree nor disagree
5 = Somewhat agree
6 = Agree
7 = Strongly agree

- In setting priorities, we must consider all groups.
- We should not push for group equality.
- Group equality should be our ideal.
- Superior groups should dominate inferior groups.
- *This is a control question to screen out random clicking. Please select "disagree" to demonstrate you have read this.*

2.1.21 National identification

Please indicate how much you agree or disagree with the following statement, using the scale.
1 = Strongly disagree
2 = Disagree
3 = Somewhat disagree
4 = Neither agree nor disagree
5 = Somewhat agree

6 = Agree
7 = Strongly agree

- I identify with the [Nationality] people.

2.1.22 Sociodemographic information

Migration experience
If you ever lived outside your country, for how long did that experience last? (please exclude vacations)

- I´ve never lived outside my country
- Less than a month
- 1-6 months
- 6-12 months
- 1-3 years
- 3-5 years
- More than 5 years

Political orientation
In politics, people sometimes talk of “left” and “right”. Where would you place yourself on this slide scale?
1 = Left
7 = Right

Age
How old are you? (Please use numbers to represent years)

Sex

- Male
- Female
- Other
- I prefer not to answer this question

Level of education
What is the highest level of education you have completed?

- Elementary school
- Junior high school
- High school
- College Associate’s degree
- College Bachelor’s degree
- Graduate/Professional degree
- I don’t know

Employment status
What is your current employment status?
o Student

- o Unemployed
- o Employed (If chosen, indicate what your profession is) _________________
- o Retired
- o Other _________________

Satisfaction with present income

Which of the descriptions comes closest to how you feel about your household's present income?

- I find it very difficult to live on the present income
- I find it difficult to live on present income
- I am managing with the present income
- I´m living comfortably on the present income
- Don't know

Perceptions about COVID-19

Please indicate your opinion using the rating scale provided.

On March 11th, 2020, COVID-19 was declared as a pandemic, a viral disease that has swept the globe. COVID-19 might be seen as a national matter, towards which [country of residence] should work alone to guarantee an effective national response, or as a global matter, towards which countries should act together to guarantee an effective global response. To what extent do you think that COVID-19 is a 'national matter' or a 'global matter'?

1 = National matter

7 = Global matter

Device

Which device are you using to fill out this survey?

- Computer
- Smartphone
- Tablet or Ipad

2.1.23 Debriefing/

THE END!

PLEASE READ THE INFORMATION BELOW

AT THE NEXT PAGE YOU WILL BE GIVEN A COMPLETION CODE TO INSERT IN CLICKWORKER PLATFORM TO ENSURE YOUR PAYMENT.

The present study is part of a PhD project "From inclusive identities to inclusive societies: Global human identification and autonomy-oriented prosocial behavior regarding immigrants", funded by Fundação para a Ciência e Tecnologia – Portugal (FCT) and being conducted at Instituto Universitário de Lisboa (ISCTE- IUL).

At the beginning, you were told that this study aims to studying how people use online platforms. However, the major goal of this project is to identify the psychosocial processes (e.g., social identification, individual characteristics) that are associated with different forms of helping and prosocial behavior regarding migrants. This is a common approach in this type of research to avoid biased responses, if the real purpose of the study was known. Your participation was very important and will help us to better understand the psychosocial processes associated with helping behaviors. You can request additional information about this study by contacting the research team: Margarida Carmona e Lima, mgfcl@iscte-iul.pt

Thank you for your participation in our study!
Please, click ">>" to get your completion code

Here is your completion code: 11S9G9PGR. Please copy the above code and paste it into the field provided within your Clickworker task form. Your Clickworker fee cannot be credited without the input of this code. If you want to let us know about your thoughts, please leave your comments below. Once you have copied this CODE, please, click ">>" to submit your responses.

2.2 Additional analysis

2.2.1 Additional information about participants in Study 2

The 224 participants were from 36 different countries, namely: England (n = 75); India (n = 43); USA (n = 21); South Africa (n = 11); Kenya (n = 9); Nigeria (n = 6); Greece (n = 5); Italy (n = 5); Philippines (n = 4); Romania (n = 4); Scotland (n = 4); Australia (n = 3); France (n = 3); Germany (n = 3); Albania (n = 2); Georgia (n = 2); Jamaica (n = 2); Russia (n = 2); Wales (n = 2); Zambia (n = 2); Algeria (n = 1); Croatia (n = 1); Czech Republic (n = 1); Estonia (n = 1); Hungary (n = 1); Indonesia (n = 1); Malaysia (n = 1); Netherlands (n = 1); Northern Ireland (n = 1); Portugal (n = 1); Serbia (n = 1); Slovakia (n = 1); Slovenia (n = 1); Spain (n = 1); Turkey (n = 1) and Vietnam (n = 1). Similar to Study 1, it is worth noting that migrants represented a minority group in terms of percentage of the total population in all countries: 40.4% of participants were living in countries where international migrants represented less than 5% of the total population (UN, 2020); 47.5% where international migrants represent 5-15%; and 12.1% where migrants represented more than 15% of the total population.

Most participants perceived their financial situation as manageable (75.4%).

2.2.2 Helping preferences: Additional analysis of the measure used in Study 2

Helping preferences: Frequencies and MCA

As in Study 1, we expected that the options a) and b) would be perceived as dependency-oriented responses and autonomy-oriented responses, respectively. To verify whether the options indeed matched with different patterns of helping responses independently of the scenario’s content, we performed a multiple correspondence analysis (MCA). The option “No help” was defined as missing values to run the MCA. The MCA revealed one relevant dimension accounting for 31.26% of the total variance, with acceptable reliability (α = .78), confirmed by discrimination measures, and revealed privileged associations within the dependency-oriented responses, as well as autonomy-oriented responses

Frequencies and MCA discrimination measures

	N	**Dim. 1**
Scenario nº 1 - **"I need to make an appointment in a health facility"**		.435
Dependency	32	
Autonomy	190	
Scenario nº 2 -**"I have an appointment with the school teacher of my children, but we don't speak a common language."**		.474
Dependency	56	
Autonomy	155	
Scenario nº 3 - **"I would like to bring my family to the new country I'm living in".**		.434
Dependency	34	
Autonomy	176	
Scenario nº 4 - **"I was a victim of discrimination."**		.070
Dependency	45	
Autonomy	167	
Scenario nº 5 - **"I need to create a CV to apply to job in the new country."**		.151
Dependency	57	
Autonomy	153	
Scenario nº 6 - **"I have a tourist visa, but I want to obtain a residence permit."**		.320
Dependency	28	
Autonomy	183	
Scenario nº 7 - **"I need to analyze my rental agreement, but I do not understand the legal standards in the new country."**		.433
Dependency	58	
Autonomy	159	
Scenario nº 8 - **"I need to write a document in the official language of the new country, which I do not speak."**		.562
Dependency	74	
Autonomy	138	
Scenario nº 9 - **"I want to meet and interact with people in the new country."**		.245
Dependency	86	
Autonomy	129	
Scenario nº 10 - **"I would like to travel across the new country."**		.270
Dependency	12	
Autonomy	208	

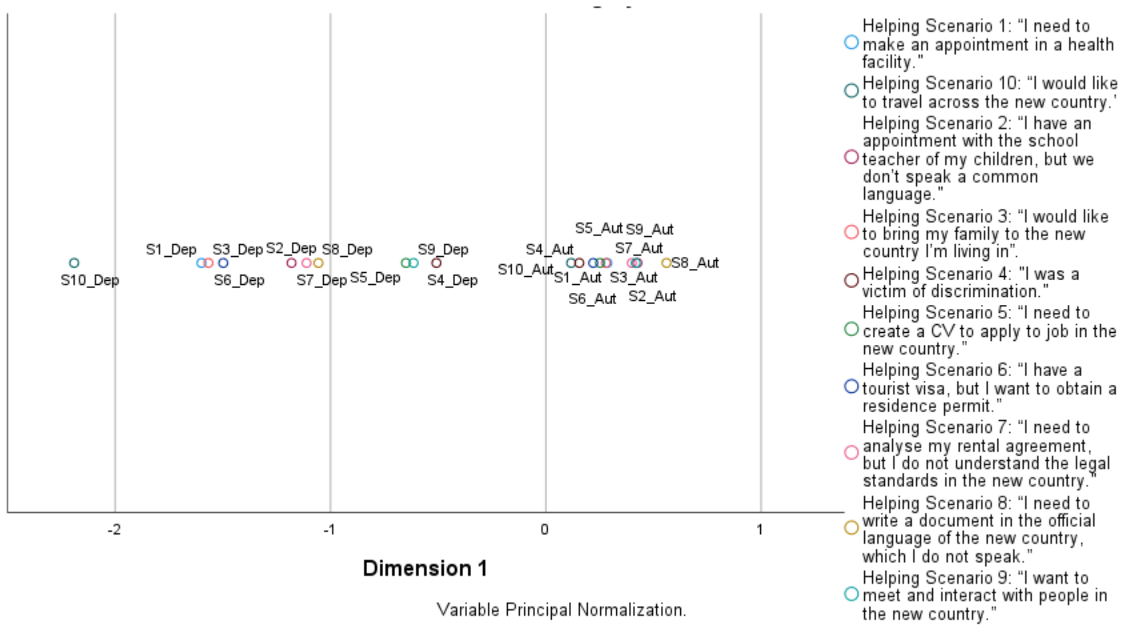

Supplementary Figure 2. Study 2: Helping preferences Scale - Topological configuration of helping preferences - Dependency-oriented and autonomy-oriented responses

2.2.3 RIP: Examining the preconditions for ingroup projection in Study 2

We first examined the preconditions for ingroup projection to occur by analyzing if the mean scores for national identification and identification with the superordinate target (control, *citizens of the world* or *humans*) were above the scale midpoint. One sample *t*-tests showed means significantly above the scale midpoint of 4 for all scales, namely national identification, $M = 5.77$, $SD = 1.28$, $t(223) = 20.779$, $p < .001$; identification with the control group of daughters and sons, $M = 5.37$, $SD = 1.64$, $t(82) = 7.649$, $p < .001$; identification with citizens of the world, $M = 5.10$, $SD = 1.59$, $t(66) = 5.696$, $p < .001$; and, identification with humans, $M = 6.22$, $SD = 1.41$, $t(73) = 13.549$, $p < .001$. These results indicate that preconditions for the occurrence of ingroup projection were satisfied.

2.2.4 Collective action and feeling towards migrants: Secondary analysis of Study 2

Means and standard deviations regarding the impact of the categories "citizens of the world" and "humans"

	**Control** (n = 83)	**C. World** (n = 67)	**Humans** (n = 74)	***Test***
	M (SD)	***M (SD)***	***M (SD)***	
**Collective action**				
Petition on civic/political rights	4.95 (1.50)	4.60 (1.49)	4.64 (1.84)	$F(4, 440) = 1.208$, $p = .306$; *Wilks'* $\Lambda = .978$, partial $\eta^2 = .01$
Petition on social/economic rights	5.25 (1.63)	5.12 (1.57)	5.34 (1.59)	
**Feelings**				
Feelings towards migrants	66.92 (22.32)	64.82 (22.24)	69.85 (21.93)	$F(2, 221) = 0.921$, $p = .400$; partial $\eta^2 = .01$

Willingness to participate in collective action

A one-way MANOVA did not reveal a significant effect of priming on participants' willingness to sign petitions advocating for migrants’ rights in the host country, neither univariate simple contrasts.

Feelings towards migrants

A one-way ANOVA did not reveal a significant effect of priming on feelings towards migrants, neither univariate simple contrasts.
